# Farmland and Cropping Pattern Dynamics in Myanmar: Implications for Food Security and Sustainable Agriculture

**DOI:** 10.3390/foods15173066

**Published:** 2026-08-29

**Authors:** Saw Yan Naing, Lin Zhen, Yu Xiao, Xingtao Liu, Xin Wen

**Affiliations:** 1 Institute of Geographic Sciences and Natural Resources Research, Chinese Academy of Sciences, Beijing 100101, China; sawyannaing16@mails.ucas.ac.cn (S.Y.N.); xiaoy@igsnrr.ac.cn (Y.X.); liuxingtao21@mails.ucas.ac.cn (X.L.); 2 College of Resources and Environment, University of Chinese Academy of Sciences, Beijing 100049, China; 3 Department of Physical & Environmental Sciences, University of Toronto Scarborough, Toronto, ON M1C1A4, Canada; xinw.wen@utoronto.ca

**Keywords:** farmland, cropping patterns, food security, sustainable agriculture, Myanmar

## Abstract

Myanmar, an agriculture-based economy, is one of the most important agricultural countries in mainland Southeast Asia. Although previous studies have documented changes in agricultural land-use and cropping patterns, trends in farmland area, cropping patterns, and food sufficiency across Myanmar’s 14 states/regions remain limited. This study addresses these gaps by using land-cover data from the global 30 m dynamic dataset and statistical data from 2000 to 2025, and household surveys from selected areas. GIS-based spatial analysis, trend analysis, and multivariate analysis of variance were applied to analyze both the direction and magnitude of farmland and cropping pattern changes over time. The results showed that Myanmar’s farmland area increased significantly (*τ* = +0.52, *p* < 0.01), expanding by 23.6% over the study period. Double cropping remained an important system, increasing by 55.9%. In the central dry zone, sufficient water availability showed the strongest effect on crop production and farmland area (*F* = 11.191, *p* < 0.001, *η*^2^*p* = 0.037), followed by soil fertility loss (*F* = 10.263, *p* < 0.001, *η*^2^*p* = 0.034). The analysis estimated that domestic consumption requirements were approximately 7.97 million tons of rice, 3.12 million tons of wheat, and 0.42 million tons of maize. These findings can support sustainable farmland use planning and inform food security policy decisions in Myanmar.

## 1. Introduction

Agriculture is the foundation of food security and rural livelihoods in most developing nations in South and Southeast Asia, contributing approximately 11% of the GDP of Southeast Asian nations in 2020 [1]. In Myanmar, agriculture accounted for more than 20% of national GDP and about 35% of total employment [2]. Amid rapid economic growth and urbanization, this sector continues to face increasing pressures from climate change, land-use conversion, and global market integration [3]. The country contains diverse agro-ecological zones, including the central dry zone (CDZ), the coastal zone, and the hilly zone [4]. These diverse agro-ecological conditions support a wide range of crop production systems, including rice, pulses, oilseeds, maize, and commercial cash crops. Rice, together with wheat and maize, is the main staple food and primary energy source for the people of Myanmar [5,6].

Food security is a multidimensional concept that involves availability, access, utilization, and stability dimensions [7,8]. The Food and Agriculture Organization of the United Nations [9] defines food self-sufficiency as the availability of domestically produced food at the national level, whereas food security encompasses not only food’s availability but also the stability of supply and people’s access to food. As a result, food self-sufficiency is widely recognized as a critical dimension of food security. Some studies from China have documented widening provincial disparities in grain’s availability despite increasing national grain output, suggesting that self-sufficiency alone is an inadequate metric for food security governance [10,11]. These dimensions of self-sufficiency also remain understudied at the subnational level in mainland Southeast Asia. According to Myint et al. [5], Myanmar needed 7.95 million tons of rice for domestic consumption. One of the main objectives of the Ministry of Agriculture, Livestock, and Irrigation is to ensure food security and food safety in Myanmar [12]. Therefore, understanding grain self-sufficiency at the state/region level is crucial.

The global farmland area has nearly doubled during the 20th century, with the greatest growth occurring in developing tropical regions [13]. There is growing evidence of both expansion and intensification in Southeast Asia [13,14]. In Myanmar, post-2010 political reforms, infrastructure investment, and trade liberalization accelerated farmland dynamics [15]. The most recent studies on changes in cropland and productivity in Central Asia and China suggested that sustainable farmland use is important for agricultural production and rural livelihood transformation [14,16]. However, comprehensive studies that integrate spatial farmland changes, cropping pattern changes, and grain self-sufficiency remain scarce. While studies globally [13] and in regions such as Central Asia and China [14,17] show that anthropogenic activity, climate change, and socioeconomic factors drive farmland and cropping pattern changes, similar state-/region-level research is still needed for Myanmar—a research gap this study aims to address.

Cropping patterns refer to the spatial and temporal arrangement of crops on agricultural land and play a critical role in determining agricultural production and food’s availability. Double cropping refers to the cultivation of two crops or the same crops twice on the same land unit within a single calendar year [18]. Mixed cropping refers to the simultaneous cultivation of two or more crops in the same field within the same growing season. Multiple cropping systems, which include double cropping and mixed cropping, are particularly important for food availability [18]. The changes in cropping patterns are crucial for promoting sustainable agriculture, which helps restore soil health and prevent soil erosion. In Myanmar, the expansion of cereal–pulse rotations has enhanced per capita caloric accessibility and dietary diversity [6,19]. However, changes in cropping patterns have been insufficiently documented at the state/region level in Myanmar. Therefore, this study also examines the cropping pattern changes at the national and regional (state/region) levels in Myanmar.

Myanmar represents a significant and policy-relevant case study in food security governance [6]. Despite historically being a rice-exporting country, the country shows strong spatial heterogeneity in domestic food production. Several states and regions face recurring grain deficits that require inter-regional transfers or external imports, while others produce substantial surpluses. These geographical disparities may influence nutritional outcomes, rural household incomes, and the sustainability of the national food system. Moreover, Myanmar is highly vulnerable to climate change, such as erratic rainfall patterns [20], cyclone risk in coastal zones [21], and drought in the CDZ [22], making evidence-based food security planning crucial.

Myanmar’s agricultural development has progressed through distinct phases. During the colonial era (1824–1948), the Ayeyawady Delta emerged as a significant rice-exporting region, a role it continues to fulfil today [23]. Following independence, Socialist-era collectivization (1962–1988) restricted agricultural investment and productivity in Myanmar [23]. The 1988 economic reforms then promoted private agriculture by expanding land allocation in the border regions of Kachin, Kayin, and Shan States [12]. After the political reforms of 2011, urban expansion and infrastructure investment accelerated, particularly in and around Mandalay and Yangon. In the meantime, land tenure reforms in the early 2010s aimed to provide smallholder farmers with more secure land tenure; however, they inadvertently led to farmland abandonment in some regions. Subsequently, the post-2021 political crisis disrupted agricultural supply chains and labor’s availability, although the impacts varied significantly by region [24].

Previous research on Myanmar’s agricultural land-use has focused on individual dimensions, such as changes in rice area and the expansion of pulse crops [25,26]. Aung Hein et al. [27] have studied agricultural land, centering on its distribution, tenure, and historical patterns of ownership, in the CDZ of Myanmar. Studies from other Southeast Asian countries have similarly examined cropland expansion and intensification [17]. However, limited studies have conducted an integrated analysis of farmland, cropping patterns, and grain self-sufficiency in Myanmar at the state/region level. To address this research gap, this study asked four questions. (1) How has farmland area changed over time and space in Myanmar? (2) How have crop production patterns changed in line with farmland change? (3) What factors explain the variations in farmland area and crop production in the CDZ? (4) What is the level of grain self-sufficiency at the national and regional levels? Four corresponding hypotheses were tested. (1) farmland area across Myanmar has experienced a statistically significant net expansion over the 2000–2025 period. (2) Cropping patterns have changed toward double cropping and commercial crops at the expense of traditional mixed cropping. (3) Agricultural resource constraint factors have greater influence on changes in farmland area and crop production than climate-related factors; however, drought and erratic rainfall significantly contribute to variations in agricultural outcomes. (4) While Myanmar has maintained an overall grain self-sufficiency surplus at the national level, significant spatial disparities exist, with the delta regions demonstrating a grain surplus and upland states facing local grain deficits.

This study presents the first nationwide assessment of farmland dynamics in Myanmar from 2000 to 2025 by integrating remote sensing data with official statistical datasets. It explores how farmland and cropping pattern changes interact with grain self-sufficiency across states and regions. In addition, combining household survey data, the study identifies key factors that influence changes in farmland area and crop production. These findings provide crucial empirical evidence to support sustainable agricultural development strategies in Myanmar and other Southeast Asian countries.

## 2. Materials and Methods

### 2.1. Study Area

**Figure A1:**
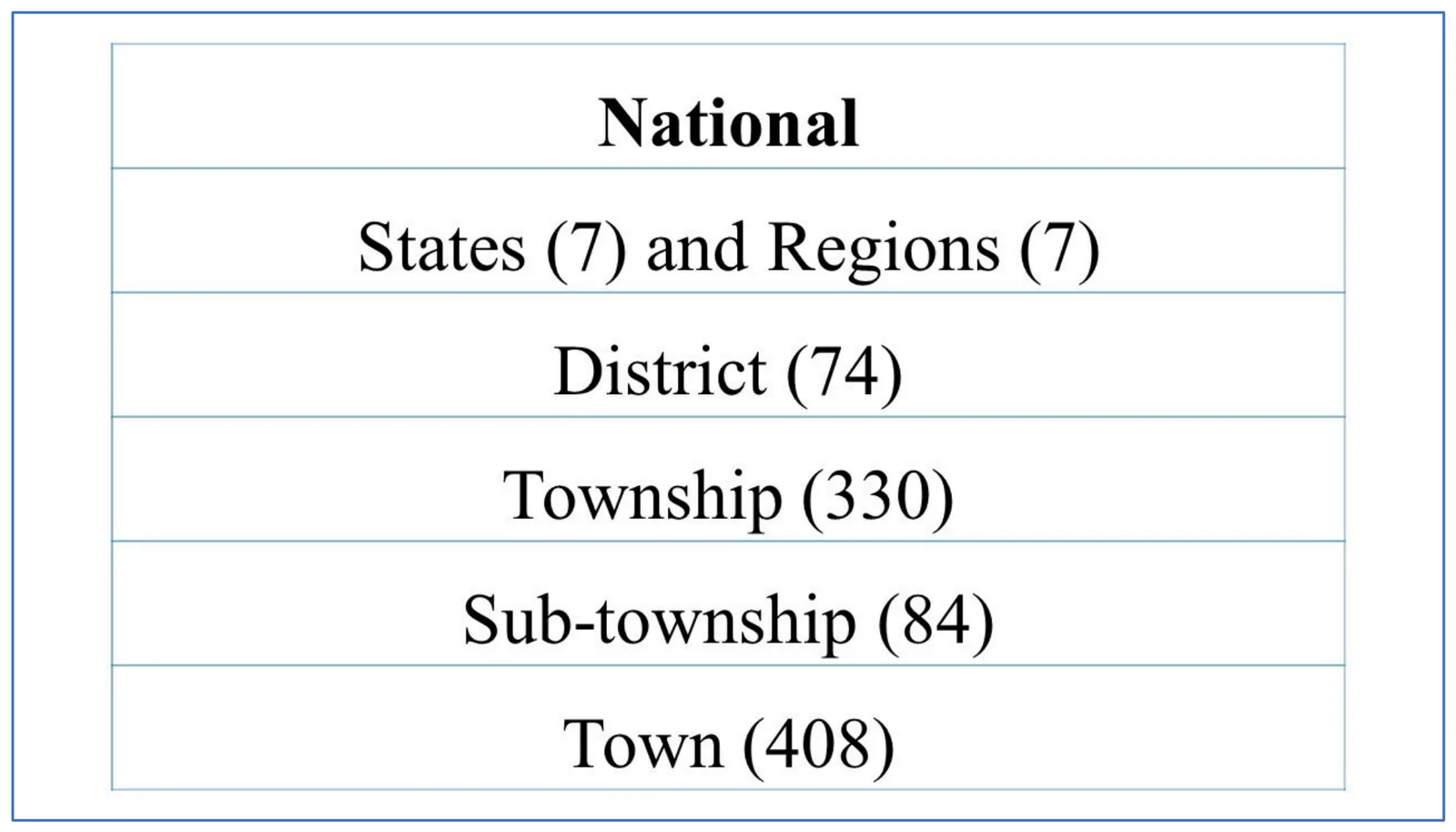
Administrative settings in Myanmar. Note: the numbers in parentheses indicate the total number of the corresponding administrative setting.

Myanmar occupies approximately 676,578 km^2^ in mainland Southeast Asia, sharing borders with China to the north and northeast, Laos and Thailand to the east, Bangladesh and India to the west, and the Andaman Sea and Bay of Bengal to the south. Myanmar’s administrative units (Figure A1) comprised states/regions, districts, townships, sub-townships, and towns [28]. Myanmar’s seven states (Chin, Kachin, Kayah, Kayin, Mon, Rakhine, and Shan) are predominantly occupied by ethnic minority populations. At the same time, seven regions (Ayeyawady, Bago, Magway, Mandalay, Sagaing, Tanintharyi, and Yangon) are geographically and culturally central to lowland Bamar majority areas. Myanmar has diverse agro-ecological conditions, ranging from the fertile lowland delta of Ayeyawady in the south to the mountainous and hilly regions of Chin, Kachin, and Shan in the north and east. The CDZ region is characterized by a dry climate and diverse farming systems that include both lowland and upland crop systems [28]. The CDZ area experiences some of the highest temperatures (40–43 °C in the dry season) and lowest annual precipitation levels (500–1100 mm/year) in Myanmar [29]. This area is about 300 m above sea level. The soil texture in the study areas includes silty clay, clay loam, and sandy loam, with a soil pH ranging from 5.0 to 8.5 and low to medium soil nutrient levels [30]. About 46% of the CDZ land area is used for agriculture, with rice, sesame, pulses, and legumes as the major upland crops [31].

### 2.2. Data Sources

This study used primary and secondary data. Farmland area data were derived from the global 30 m land-cover dynamic dataset (the GLC_FCS30D dataset, https://zenodo.org/records/15063683 (accessed on 1 November 2025)) [32], which provides annual land-cover maps from 1985 to 2022. This dataset achieved an overall accuracy rate of 80.88% across the 10 major land-cover types. The “farmland” class was extracted and clipped to each administrative unit, and farmland area was calculated for each unit using the Zonal Statistics tool. Administrative boundary shapefiles for Myanmar’s 14 states/regions were obtained from the Myanmar Information Management Unit [33]. The extracted data were subsequently cross-checked against official statistical records (Supplementary S1). To analyze farmland area trends from 2000 to 2025, additional data were also obtained from the Myanmar Central Statistical Organization’s statistical yearbooks.

The national and regional crop production and cultivated area data from 2000 to 2025 were used to identify cropping patterns (Supplementary S2 and S3), which were sourced from the Myanmar Central Statistical Organization’s statistical yearbooks https://www.csostat.gov.mm/ (accessed on 16 January 2026) [2]. While statistical yearbooks up to 2024 are available as open-access resources, the Myanmar Statistical Yearbook for 2025 was obtained from the Department of Agriculture through official institutional communication because it had not yet been released online at the time of analysis. To ensure data reliability, the 2025 data were cross-checked against historical trends and reports published by the Ministry of Agriculture, Livestock, and Irrigation.

To analyze the factors influencing changes in farmland and crop production, primary data were collected via structured questionnaires administered across Yamethin and Ottara in the CDZ of Myanmar. Given that a nationally representative household survey was financially unfeasible, survey sites were selected in view of the following criteria.

- Yamethin District: chosen for the use of mixed cropping systems and increasing market-oriented production features of the farmers;
- Ottara District: chosen for its heterogeneity in agricultural production systems, upland and lowland cultivation, and market access.

This study adopted a multistage sampling strategy. At the first stage, districts were purposively selected to represent different agricultural production systems [34]. At the second stage, a simple random sample of farm households was selected from the identified villages [35]. Village lists were obtained from the respective townships’ administration offices. Farm households were selected using simple random sampling. Six villages were selected from each district, with 50 farm households sampled per village. In total, 603 farm households were surveyed using a structured questionnaire; of these, 600 complete responses were included in the final analysis.

This sample size meets the minimum requirement for achieving statistical representativeness. According to Yamane’s sampling, a minimum sample of 398 respondents is adequate to obtain a 95% confidence level with a 5% margin of error in the population of 131,851 households in Yamethin and Ottara Districts [2,36]. Data were collected through six steps:

- Obtaining administrative authorization from township officers;
- Obtaining approval from local village leaders;
- Conducting preliminary field observations;
- Validating selected indicators with five experts (two from the University of Chinese Academy of Sciences, one agricultural economist from Yezin Agricultural University, and two agricultural officers from the Department of Agriculture);
- Training field enumerators; and
- Conducting farm household interviews.

Data collection was conducted using face-to-face interviews with farmers in each village from January to March 2026. A total of 6 enumerators were trained for data collection. Before the survey, a pilot survey was conducted in January involving 28 non-sample farm households to ensure logical flow and consistency.

This study was conducted in accordance with the regulations of the Ethics Committee at the Institute of Geographical Sciences and Natural Resources Research, Chinese Academy of Sciences. Under the “Measures for Ethical Review of Life Sciences and Medical Research involving Humans” issued by the State Council of China, this study does not require ethical review. Moreover, standard ethical protocols were prioritized throughout the field survey by obtaining informed consent, maintaining participants’ anonymity, and respecting local cultural values. More specifically, all participants were informed that the survey questions were related to climate change impacts on crop production and farming adaptation strategies. The structured questionnaire included the following: (i) farm household characteristics, (ii) landholding and cropping patterns, (iii) climate events and impacts, (iv) perceived constraints and influencing factors, and (v) climate change adaptation strategies. Please see Supplementary S4 Farmer Questionnaire for the details. The questionnaire survey was conducted anonymously. The questionnaire collected basic demographic characteristics, including household identification code, gender, age, education, farming experience, family size, and household labor, for research purposes. No directly identifying personal information, such as the participants’ names, telephone numbers, or national identification numbers, was collected. The household identification code was used solely for data management and was not linked to the participants’ names or other direct identifiers. Furthermore, the participants were assured that all collected data would be used solely for academic research purposes and that participation involved no potential risks. All participants demonstrated full understanding and support for the study.

Data on grain yield and urban and rural populations (Supplementary S5) were obtained from the Myanmar Statistical Yearbook. Grain consumption data were calculated using population data. Per capita food consumption parameters were obtained from published research and international databases. Drawing on the literature on Myanmar, international databases, and our field survey experience, FAO standard parameters are suitable for Myanmar, which allows for comparability on a regional and global scale [37]. According to FAO guidance, total cereal consumption was assumed to be 208.8 kg per person per year in urban areas and 230.8 kg per person per year in rural areas. Rice consumption was stratified by urban (133 kg/year) and rural (165 kg/year) populations [5]. Maize consumption was specified at 8.2 kg/person/year for both urban and rural populations [38]. Wheat consumption was assumed to be 67.6 kg/year for urban and 57.6 kg/year for rural populations [39]. These data were used to analyze grain self-sufficiency in the states/regions of Myanmar.

### 2.3. Analytical Methods

#### 2.3.1. Farmland Change Detection

Trend analysis and compound annual growth rate methods were used to evaluate farmland area in Myanmar [40,41,42]. Absolute area changes and proportional changes relative to the 2000 baseline were calculated for each of the 14 states/regions. These methods are widely applied in agriculture and land-use change studies to measure the direction and magnitude of long-term changes [23].

#### 2.3.2. Cropping Pattern and Crop Production Change Analysis

Cropping pattern change was calculated using the percentage-change method, which measures the change in the cultivated area of crop types between two reference years [18]. This approach was applied over five-year intervals and across the full 2000–2025 period for the major crop groups: grains (rice, wheat, maize), oilseeds (groundnut, sesame, sunflower), pulses (black gram, green gram, soybean, pigeonpea, lablab bean, chickpea), and other crops (potato, cotton, sugarcane). Crop production changes were classified into five levels: significant increase (>+25%), moderate increase (+10% to +25%), stable (−10% to +10%), moderate decline (−10% to −25%), and significant decline (<−25%).

In addition, this study employed multivariate analysis of variance (MANOVA) within a general linear model framework to examine the simultaneous effects of farmers’ perceptions of climate-related factors and agricultural resource constraint factors on farmland area and crop production. The dependent variables were farmland area (ha) and crop production (ton/ha) by the sampled farmers (Table A1). Nine binary independent variables (1 = yes, 0 = no) were considered into two key theoretical dimensions:

- Climate-related factors: climate change, drought, floods, and erratic rainfall;
- Agricultural resource constraint factors: sufficient water availability, soil fertility loss, limited access to quality seed, labor scarcity, and lack of market-based incentives.

MANOVA was selected because the dependent variables represent related agricultural outcomes that may be jointly supported by common environmental and socioeconomic drivers. Multivariate effects were evaluated using Pillai’s Trace statistics, followed by univariate tests of between-subjects effects to identify factor-specific influences on crop production and farmland area.

The assumptions of MANOVA were evaluated before conducting the analysis. The three main assumptions are that the observations are independent; the dependent variables have a multivariate normal distribution within groups; and the variance–covariance matrices are homogeneous across groups [43]. Normality was evaluated using the standardized residuals of the dependent variables within groups defined by the binary independent variables. Residual normality was examined using histograms, Q–Q plots, skewness, and kurtosis statistics. The homogeneity of the covariance matrices between groups was assessed by Box’s M test, while equality of error variances for each dependent variable was evaluated by Levene’s test. Because Pillai’s Trace is robust to moderate violations of multivariate normality and variance–covariance assumptions, it was selected as the primary multivariate statistic when minor deviations from these assumptions were observed.

#### 2.3.3. Grain Self-Sufficiency Indicator

Rice is the primary staple food in Myanmar, contributing approximately 51% and 62% of calories consumed in urban and rural households, respectively [44]. Together, rice, wheat, and maize were considered to be the components of the grain self-sufficiency indicator in this study.

The grain self-sufficiency rate, following approaches widely applied in regional food security assessments [10,11], was calculated using the following formula

*R_it_* = *P_it_*/*C_it_* (1)

where *R_it_* is the grain self-sufficiency rate in region or country *i* at time *t*, *P_it_* is the total grain production in state/region *i* at time *t*, and *C_it_* is the estimated grain consumption in state/region *i* at time *t*. Based on the self-sufficiency rate *R_it_*, three categories were defined: (i) surplus (*R_it_* > 1.0); (ii) sufficiency (*R_it_* = 1.0); and (iii) deficit (R_it_ < 1.0) [16].

Grain consumption in urban and rural areas was calculated as

*G_it_* = (*f_it_rice_* + *f_it_maize_* + *f_it_wheat_*) × *Po_it_* (2)

where *G_it_* is the urban or rural grain consumption in time *t* in area *i*; *f_it_rice_*, *f_it_maize_*, and *f_it_wheat_* are urban or rural rice consumption per capita, maize consumption per capita, and wheat consumption per capita, respectively; and *Po_it_* represents the population of time *t* in area *i*.

Total grain consumption in area *i* of year *t* is defined as

*G_total_it_* = *G_uit_* + *G_rit_* (3)

where *G_total_it_* refers to the total grain consumption in area *i* of year *t*, *G_uit_* is urban grain consumption in area *i* of year *t*, and *G_rit_* represents rural grain consumption in area *i* of year *t*.

#### 2.3.4. Spatial Analysis

ArcMap 10.8 software was used to map the spatial distribution of crop production and grain self-sufficiency ratios across Myanmar’s states/regions for each five-year reference year. The state-/region-level grain production and self-sufficiency rate were tabulated in Excel, then joined to the polygon shapefile attribute table using the administrative unit name as the unique key. The maps were generated to visualize patterns and to identify the persistent surplus, sufficiency, and deficit states/regions for each reference year.

## 3. Results

### 3.1. Farmland Changes at National and Regional Levels

**Figure 1:**
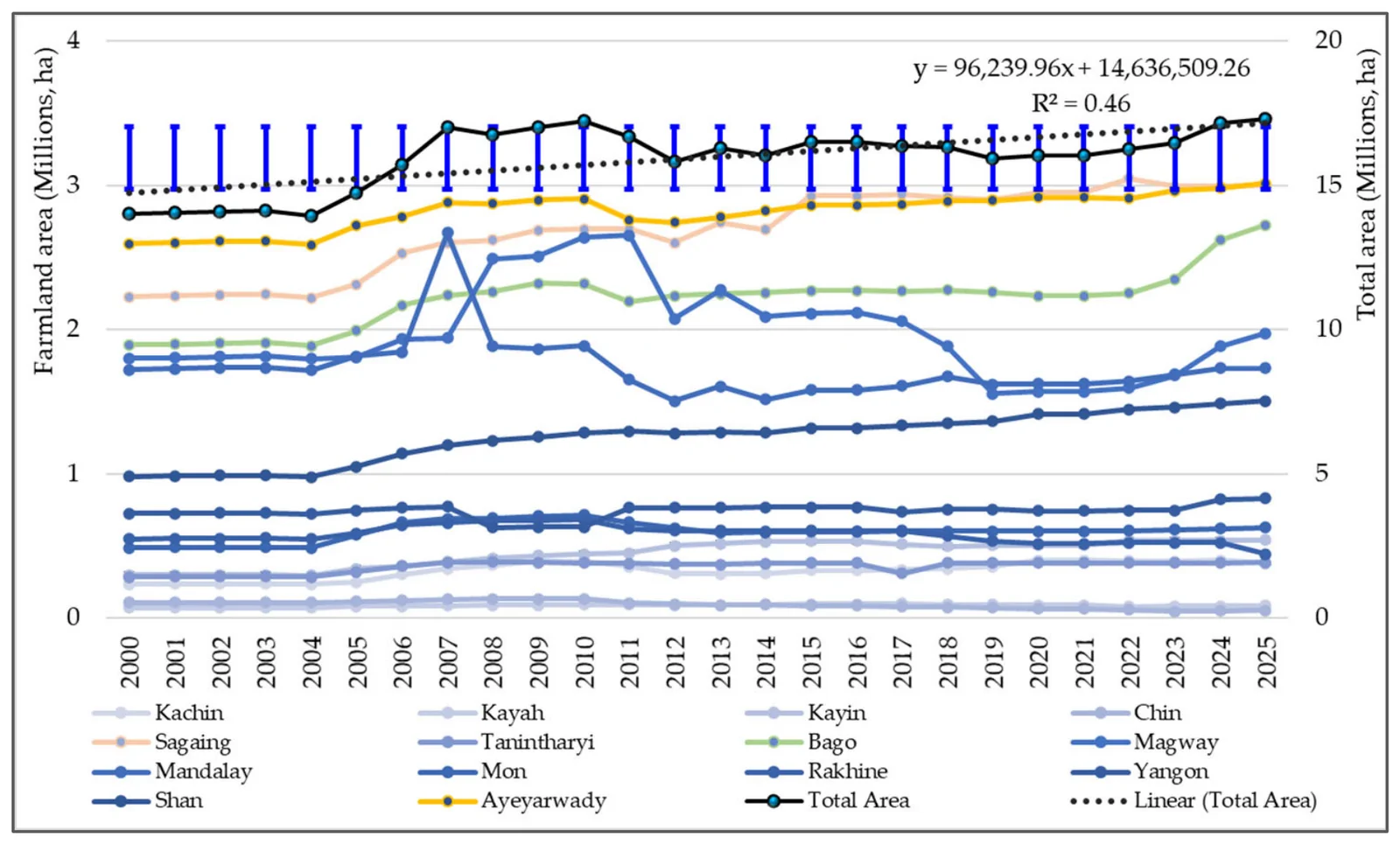
Trends of farmland area in Myanmar from 2000 to 2025. The black dotted line represents the linear trend of total national farmland area. The orange line shows the trend for Ayeyawady Region. The blue vertical error bars indicate the standard deviation of total farmland area.

The Mann–Kendall test on Myanmar’s farmland area revealed a statistically significant positive trend (*τ* = +0.52, *p* < 0.01). Myanmar’s total farmland area showed an overall increasing trend from 13.99 million hectares (Mha) in 2000 to 17.30 Mha in 2025, a net increase of +23.6% (Figure 1). This study identified three distinct phases of farmland area change in Myanmar. The first phase (2000–2010) was a period of rapid growth, with total farmland expanding from 13.99 Mha to 17.22 Mha, a net increase of +23.1%. This was followed by the second phase (2010–2019), during which the national farmland area declined by −7.6%, influenced by land degradation, cyclone-induced agricultural land loss, and cropland abandonment. A third phase (2019–2025) recorded farmland recovery of +8.8%.

The Mann–Kendall test revealed significant increasing trends in farmland area across most states and regions of Myanmar between 2000 and 2025 (Table 1). The positive trends were observed in Kayin, Shan, Sagaing, and Ayeyawady. On the other hand, significant declining trends were found in Rakhine and Chin. The coefficient of variation ranged from 0.04 in Ayeyawady to 0.28 in Chin, indicating significant regional differences in the temporal stability of farmland area. Magway and Mandalay showed statistically non-significant trends, suggesting relatively stable farmland extents during the study period.

Ayeyawady Region consistently maintained the largest farmland area (CAGR +0.60%, *M*–*K τ* = 0.73). Sagaing Region ranked second, showing an increase of +34.63% and a consistently positive trend across all three phases. Bago Region recorded prominent growth, a net increase of +43.88%, supported by the intensification of the rice–pulse cropping pattern. The findings show that Ayeyawady, Sagaing, and Bago were the agricultural heartland of the country.

In contrast, Chin State lost −50.09% of its farmland (M–K τ = −0.66, *p* < 0.01), reflecting the mountainous area and inadequate irrigation access. Rakhine State also showed a declining trend, decreasing by −19.42%. Meanwhile, Kayin State showed an increasing trend, a net increase of +80.9% (M–K τ = 0.81, *p* < 0.01). Chin and Rakhine States were low-production areas in Myanmar, indicating that improvements in crop productivity and agricultural technologies may be needed.

The findings show that double cropping remained the dominant cultivation practice in Myanmar (Table 2). Areas under double cropping increased from 3.74 Mha in 2000 to 5.83 Mha in 2025, an increase of +55.88%. Areas generally increased between 2000 and 2010, declined during 2015–2020, and subsequently recovered by 2025. Areas under mixed cropping recorded a fluctuating trend in Myanmar. They increased from 1.19 Mha in 2000 to a peak of 3.86 Mha in 2010 before declining to 1.51 Mha in 2025. These findings indicate that double cropping accounted for the largest proportion of cultivated area during the study periods, while mixed cropping showed greater inconsistency.

### 3.2. Cropping Pattern Changes at National and Regional Levels

Table 3 shows the changes in cultivated areas for major crops in Myanmar between 2000 and 2025. Rice remained the dominant cereal crop throughout the study period, accounting for the largest cultivated area in Myanmar. Similarly, maize recorded an increasing area of cultivation among grain crops, particularly in Shan, Kachin, and Kayin States. In contrast, areas of wheat cultivation declined substantially. These changes reflect a broader shift in cropping patterns toward commercial crops such as maize at the expense of traditional staples such as wheat.

Areas for both oilseed and pulse crops showed significant growth in Myanmar. Among the oilseed crops, groundnut cultivation showed the greatest expansion, having more than doubled its cultivated area between 2000 and 2025. Sesame area increased moderately, while sunflower area represented an overall decline at the national level. The pulse crops expanded, with particularly large increases observed in chickpea, green gram, and black gram. Areas for soybean, pigeonpea, and lablab bean also increased, although at lower rates.

At the regional level, areas of maize cultivation increased in several states and regions, particularly Kayin, Yangon, Bago, and Shan (Table 4). The results show that areas of pulse cultivation expanded in most regions, with the largest increases recorded in Kayin, Tanintharyi, and Bago. Areas of oilseed cultivation also increased across many regions, especially in Bago, Ayeyawady, and Sagaing. In contrast, rice area decreased in several western and central regions, particularly Mandalay, Chin, and Tanintharyi. These dynamics highlight an evolving regional cropping landscape characterized by an increased allocation of farmland to commercial crops like maize, pulses, and oilseed crops, alongside a local reduction in rice. The cropping pattern changes varied among regions, demonstrating the differences in agricultural land-use change across the country during the study period.

### 3.3. Crop Production Changes at National and Regional Levels

**Figure 2:**
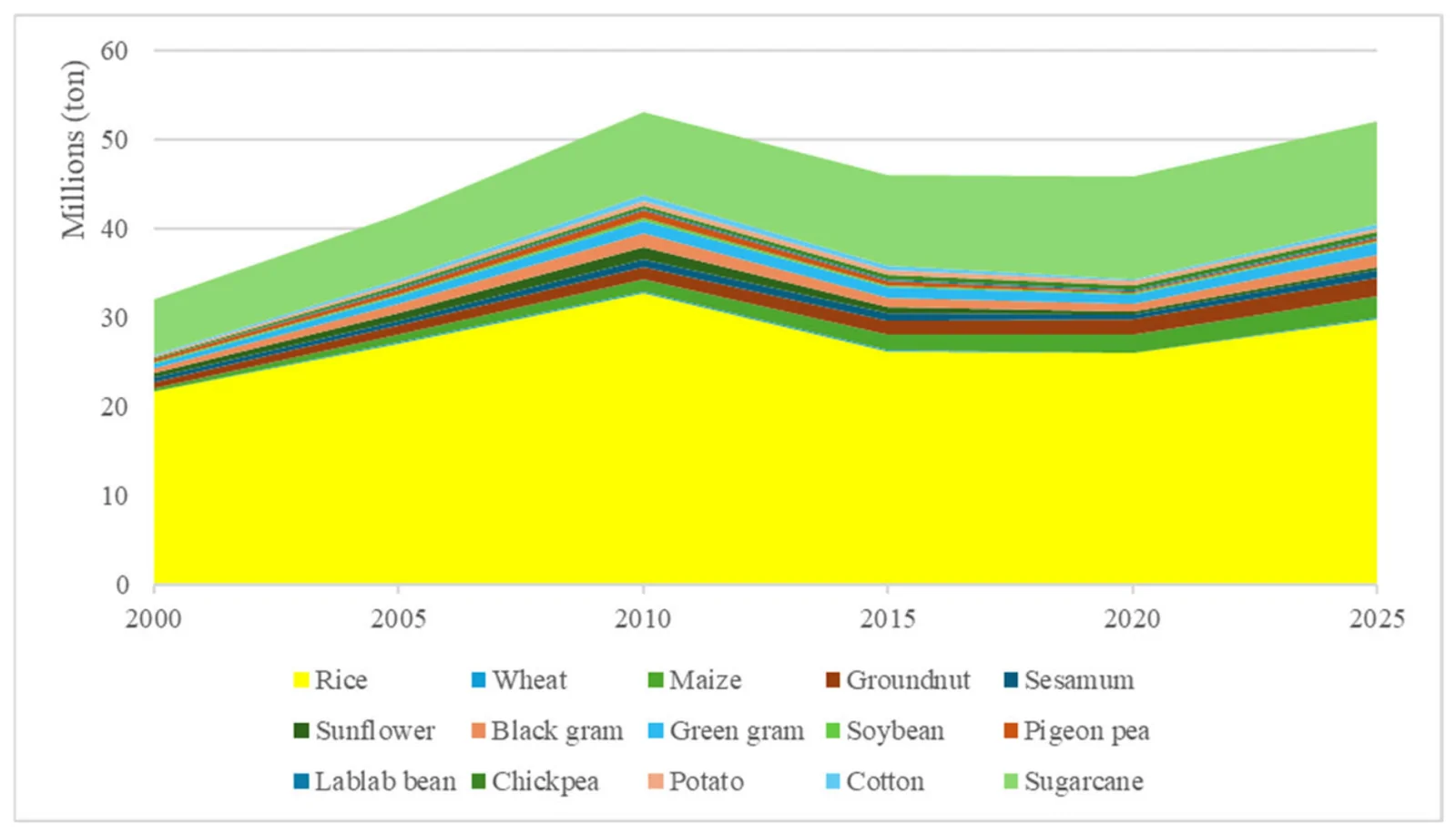
Trend of dominant/main crop production in Myanmar from 2000 to 2025.

Between 2000 and 2025, total grain production increased over the study period, driven by increases in rice and maize production in Myanmar (Table 5). Myanmar’s rice remained the dominant crop, with production reaching its highest in 2010 before declining and recovering by 2025 (Figure 2). The results show that the 2010 peak coincided with the maximum farmland expansion and intensification of double cropping.

Maize recorded the most notable production growth among grain crops, increasing more than fivefold during the study period. Wheat production changed relatively little despite fluctuations in the cultivated area. Among the oilseed crops, groundnut and sesame production have increased steadily, while sunflower production, another important oilseed crop, showed an overall downward trend over the past 25 years.

Figure 2 also indicates that pulse production grew steadily in Myanmar. The largest proportional increases were observed for chickpea, lablab bean, black gram, and green gram. Soybean and pigeonpea showed comparatively smaller increases. Among the other crops, sugarcane production nearly doubled over the study period. Cotton production increased with temporal fluctuations in Myanmar. Potato production increased moderately relative to its 2000 production level.

**Figure 3:**
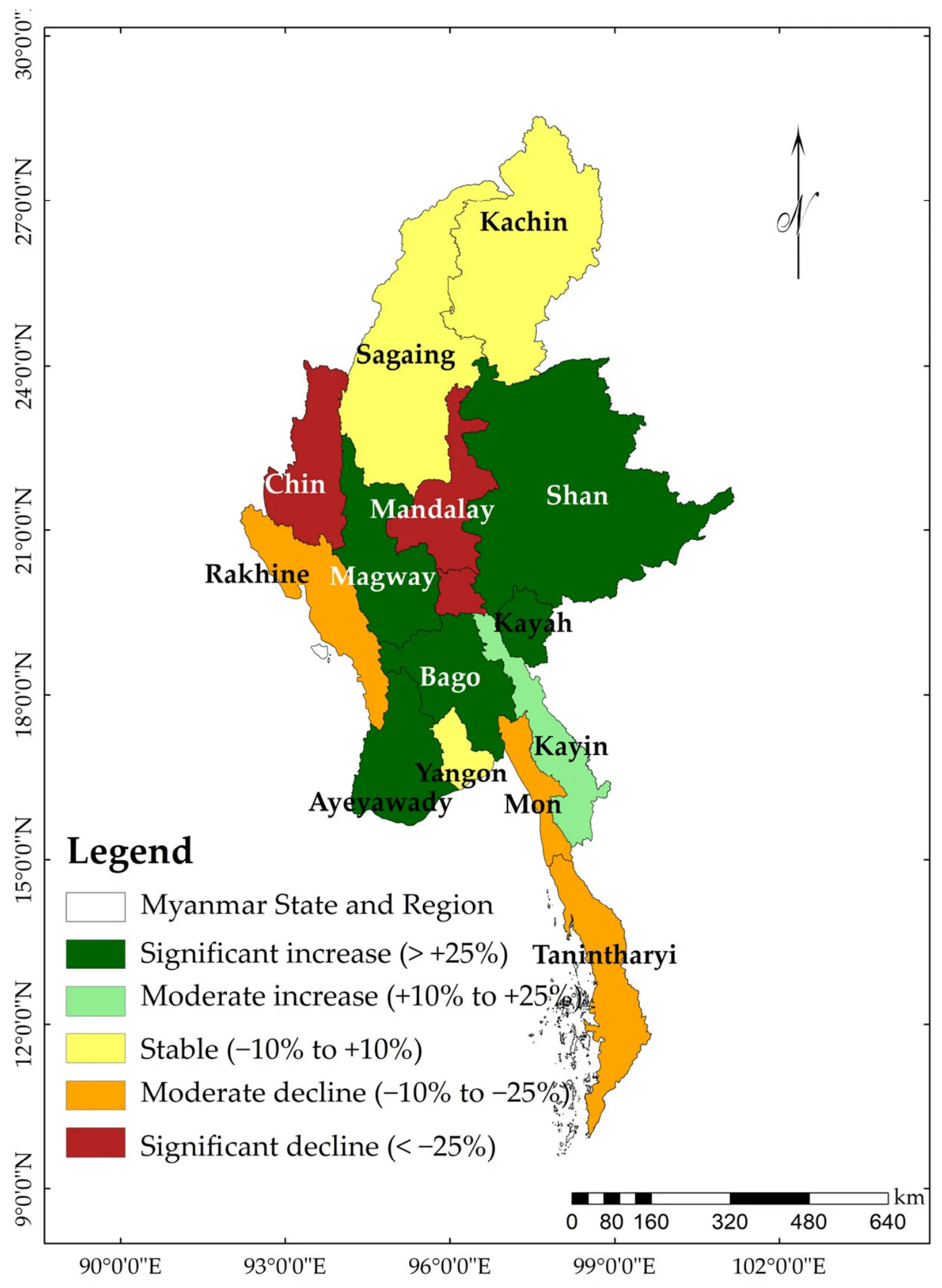
Crop production changes in the states/regions of Myanmar from 2000 to 2025.

Figure 3 illustrates the spatial pattern of change in crop production in Myanmar from 2000 to 2025. Lowland delta regions (Ayeyawady, Bago, Yangon) showed net increases in rice and pulse production, supported by good irrigation systems and crop yield improvements. Crop production in Ayeyawady and Bago increased significantly, but remained stable in Yangon. The CDZ (Magway, Mandalay, Sagaing) recorded area expansion and diversification into oilseeds, pulses, and cotton, reflecting market incentives. Magway showed a significant increase in crop production, while Sagaing experienced stable crop production. Hilly regions (Shan, Kachin) demonstrated specialization in maize, potato, and other upland crops. A significant decline in production was concentrated in Chin and Mandalay.

**Figure 4:**
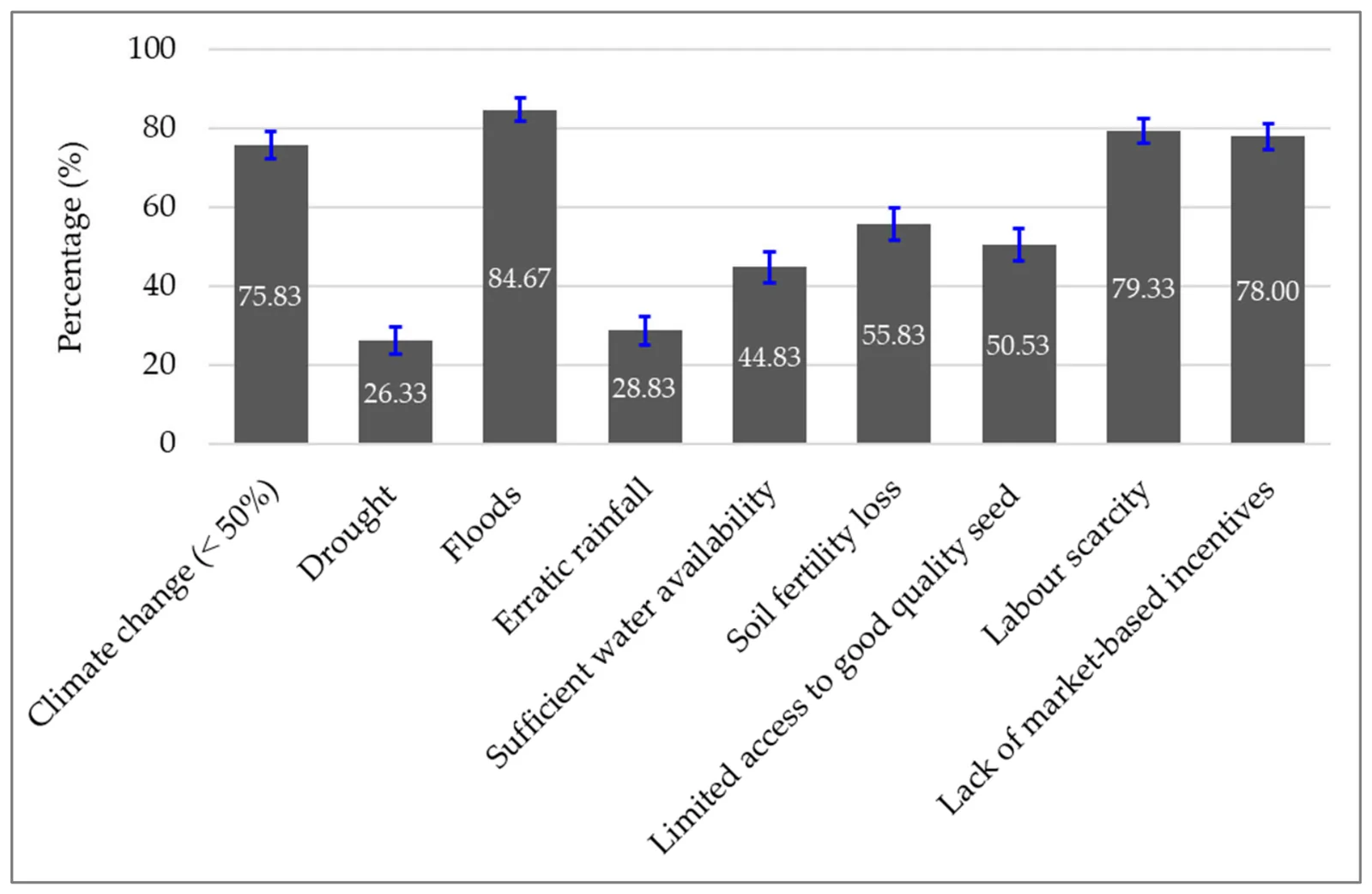
Percentage of respondents reporting each influencing factor (*n* = 600). Error bars represent 95% confidence intervals.

Figure 4 shows farmers’ perceptions of factors that explain variations in farmland area and crop production in the CDZ of Myanmar. Flooding was reported by the highest proportion of respondents (84.67%); however, there it was not significantly associated with the combined variation in farmland area and crop production (*p* = 0.383). Labor scarcity (79.33%) and lack of market-based incentives (78.00%) were also reported by farmers but were not statistically significant (*p* = 0.538 and *p* = 0.712).

The MANOVA results show that drought, erratic rainfall, sufficient water availability, and soil fertility loss significantly affected the combined variation in crop production and farmland area (Table A3). The strongest multivariate effects were observed for sufficient water availability (*F* = 11.191, *p* < 0.001, *η*^2^*p* = 0.037) and soil fertility loss (*F* = 10.263, *p* < 0.001, *η*^2^*p* = 0.034). Soil fertility loss had the largest effect on crop production (*F* = 20.502, *p* < 0.001, *η*^2^*p* = 0.034), whereas water availability was significantly associated with both crop production and farmland area (Table A4).

For the assumptions of MANOVA, Box’s M test indicates no significant violation of covariance homogeneity (Box’s M = 308.206, *F* = 1.151, *p* = 0.075), suggesting that the covariance matrices of the dependent variables were equivalent across groups (Table A2). Levene’s test shows homogeneous error variances for crop production (*p* = 0.226), although farmland area indicates some variance heterogeneity (*p* = 0.014). Given the large sample size and the robustness of Pillai’s Trace statistics, the MANOVA results were considered to be reliable.

### 3.4. Grain Self-Sufficiency at National and Regional Levels

**Figure 5:**
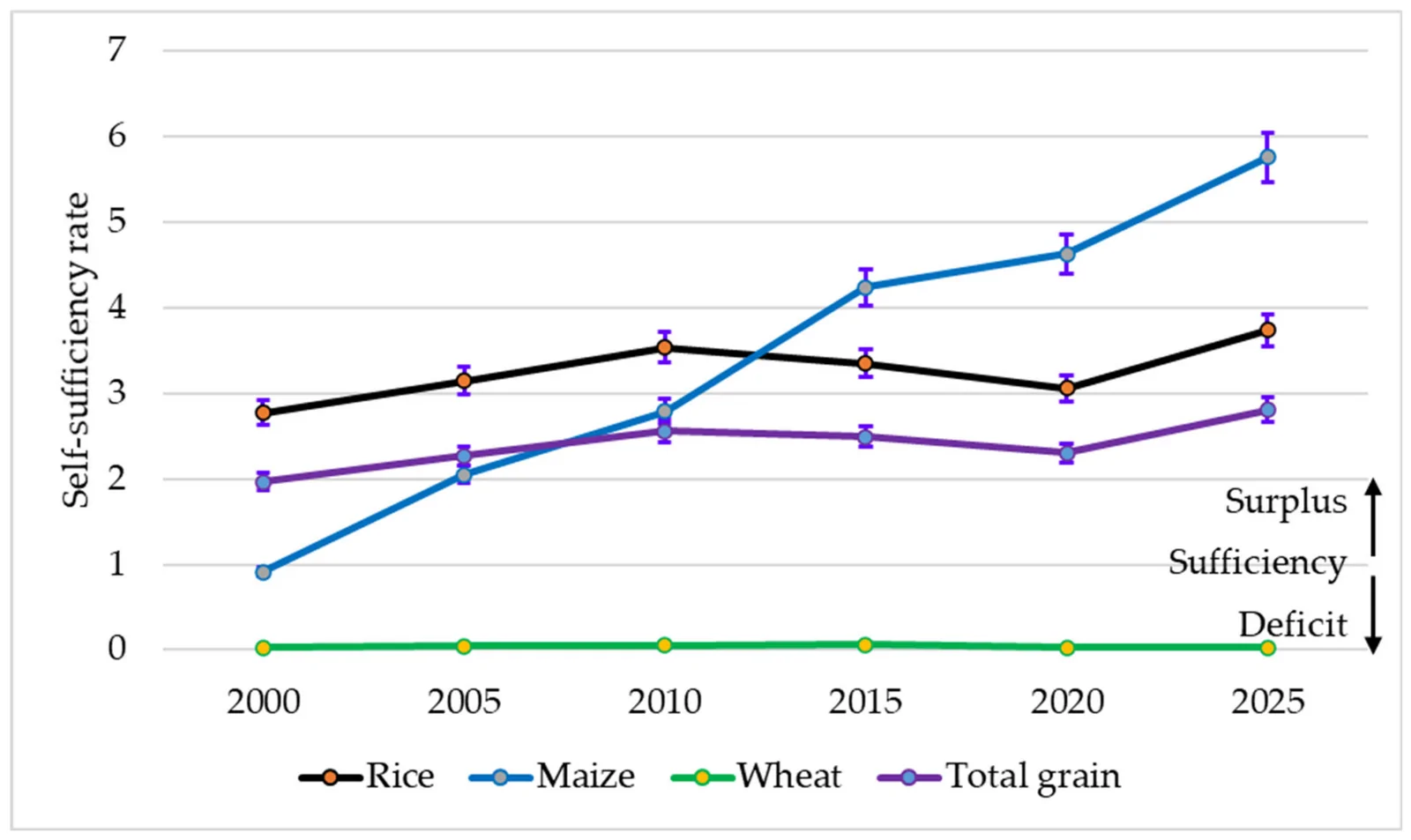
Staple food self-sufficiency at the national level in Myanmar from 2000 to 2025.

**Figure 6:**
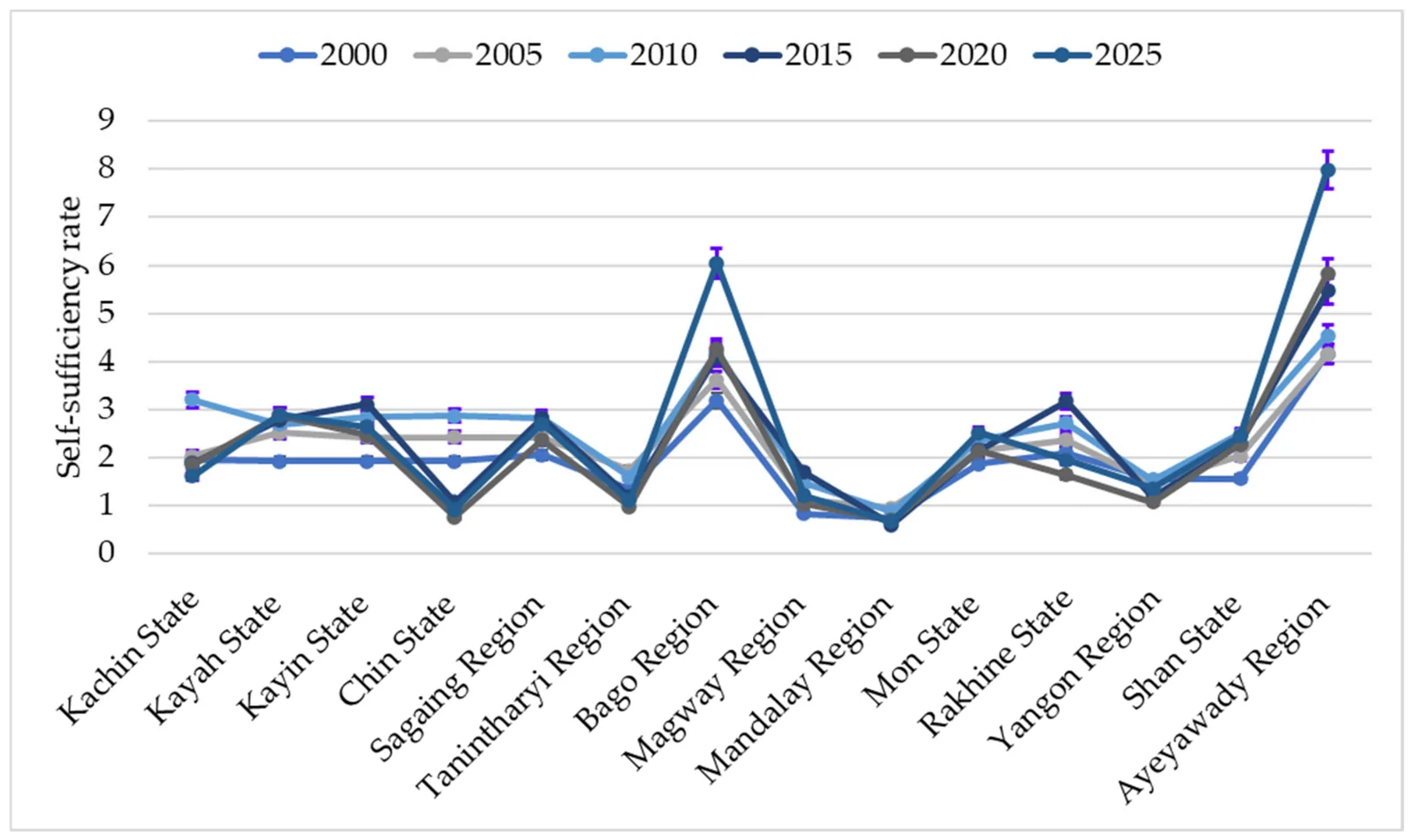
Staple food self-sufficiency at the regional level in Myanmar from 2000 to 2025.

**Figure 7:**
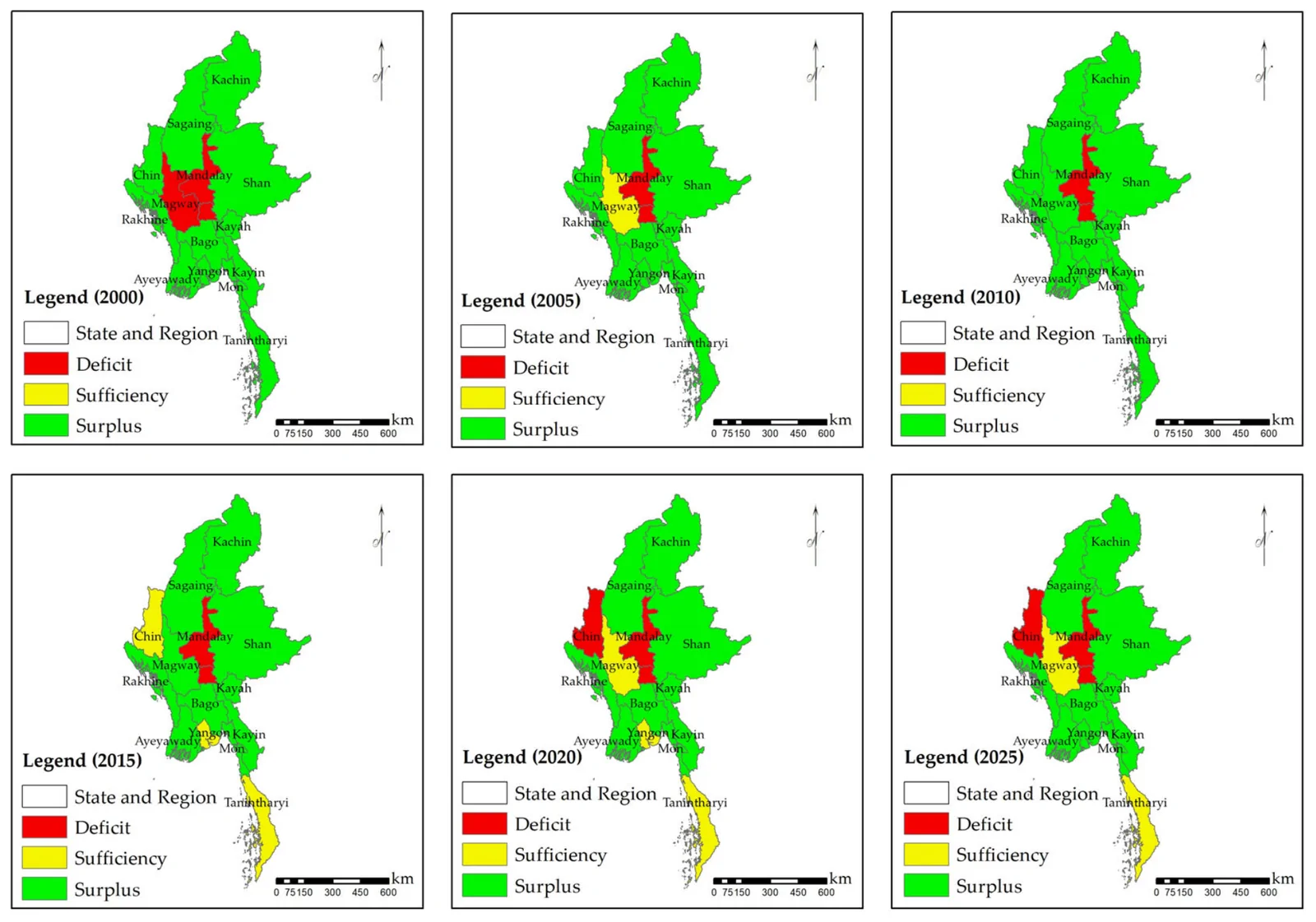
Spatial distribution of staple food self-sufficiency across Myanmar’s states/regions from 2000 to 2025.

Based on the projected urban and rural population in 2025, the results show that domestic consumption requirements are estimated at 7.97 million tons of rice, 3.12 million tons of wheat, and 0.42 million tons of maize (Supplementary S4). At the national level, Myanmar sustained a positive grain self-sufficiency rate throughout the entire study period (Figure 5). The total grain self-sufficiency rate increased from 1.97 in 2000 to 2.81 in 2025. This trend aligns with the rice self-sufficiency rate, the expansion and shrinkage phases observed in farmland area, and crop production. The results confirm that total staple food production capacity remained above domestic consumption needs throughout the study period. Ayeyawady Region recorded the highest grain self-sufficiency rate (7.99 in 2025) in Myanmar (Figure 6 and Figure 7). Bago Region also showed an increasing grain self-sufficiency rate during the study period. In contrast, Rakhine State showed a decreasing grain self-sufficiency rate from a high ratio of 3.18 in 2015 to 1.95 in 2025, suggesting that farmland area losses and production decline are diminishing its surplus capacity. Magway, Tanintharyi, and Yangon Regions remained slightly above the grain self-sufficiency rate. In contrast, Mandalay Region and Chin State failed to achieve grain self-sufficiency, due to significant farmland area reductions and limited opportunity for intensification, given their mountainous topography.

## 4. Discussion

### 4.1. Farmland Expansion and Agricultural Intensification

Myanmar experienced a net expansion of farmland between 2000 and 2025, consistent with regional patterns across Southeast Asia [17,45]. More specifically, irrigation development and multiple cropping systems have intensified agricultural production in Vietnam, while agricultural intensification driven by mechanization and the development of agricultural technology have increased crop specialization in Thailand [17]. Similarly, Cambodia and Laos have experienced substantial expansions of maize and commercial crop production, often associated with regional trade and cross-border investment. Moreover, Qin et al. [14] reported that the average cultivated farmland footprint for food consumption decreased by 56% due to improvements in agricultural technologies and cropping systems in Southeast Asia.

Previous studies in Myanmar have demonstrated that farmland area increased from 9.5% in 2010 to 18.15% in 2020 [46]. The political reforms after 2011 opened up international trade, and export products were concentrated in the agriculture sector, leading to agricultural intensification. Belton et al. [47] also reported that Myanmar has experienced a rapid expansion of agricultural mechanization since 2011, which may have contributed to farmland expansion and agricultural intensification. Land tenure reforms introduced in the early 2010s, along with land governance, influenced farmland expansion at both the regional and national levels [48]. However, Takeshima et al. [49] indicated that the interaction of climate change and agricultural intensification has influenced agricultural development differently in Myanmar.

These findings suggest that future agricultural policies in Myanmar should adopt region-specific approaches rather than relying on uniform national strategies. Strategic investment in irrigation infrastructure, climate-smart agriculture, sustainable land management, and secure land tenure systems are critical for achieving sustainable agricultural intensification.

### 4.2. Cropping Pattern Changes and Their Food Security Implications

The results revealed significant changes in cropping patterns, with the expansion of maize production emerging as a notable trend. Maize production is mainly driven by China’s demand for livestock feed. Contract farming arrangements have encouraged farmers in upland regions to shift from traditional subsistence farming to commercial maize production [50]. Likewise, maize expansion has been associated with regional markets and border trade in Laos, Thailand, and upland Vietnam [14]. The expansion of irrigated farming and the increasing adoption of agricultural machinery have facilitated the growth of double and multiple cropping systems.

The growth of pulses’ crop area and production has beneficial food security implications. Pulses are important sources of dietary protein, iron, and zinc, and their inclusion in cereal rotation systems improves soil fertility via biological nitrogen fixation, reducing synthetic fertilizer needs [51]. Myanmar’s emergence as a leading global pulse exporter (1.36 Mt in 2019, ranking third worldwide) has allowed export-oriented pulse production to generate foreign exchange and increase farm incomes [52]. However, if consistently high export prices prevent domestic consumers from accessing pulses at affordable prices, the dietary benefits may not be realized domestically. This tension illustrates the challenge that global commodity market integration poses for national food security governance [53].

The expansion of maize and pulse production presents both opportunities and challenges. Increased commercialization can enhance farm incomes and stimulate rural economic growth. However, excessive dependence on export-oriented commodity production may increase vulnerability to international market fluctuations while simultaneously threatening environmental sustainability.

The observed decline in mixed cropping systems is particularly concerning because diversified farming systems provide important ecosystem services, such as soil erosion control and soil fertility enhancement. This decline may indicate increased vulnerability in Myanmar’s agricultural systems, as reduced agricultural diversity lowers resilience to climatic and economic shocks [54]. In contrast, the rise of double cropping in irrigated zones indicates agricultural intensification that has positive implications for crop production per unit area [3,55]. Consequently, promoting sustainable intensification, crop diversification, conservation agriculture, and integrated farming systems can help balance agricultural productivity growth with long-term environmental sustainability.

Although floods were the most frequently reported climate-related factor, their statistical effect on production was insignificant. This inconsistency may result from differences between perceived vulnerability and measured production impacts. Farmers often evaluate risks on the basis of memorable extreme events [56], livelihood disruption, and potential losses rather than average annual production outcomes. In the CDZ, floods are relatively less frequent than drought events, and their impacts may be concentrated in specific locations or agricultural seasons.

Furthermore, farmers may adopt climate change adaptation strategies, such as adjusting planting dates, using resistant varieties, and crop diversification, which may reduce crop production losses [57]. Therefore, flood perception represents an important indicator of farmers’ climate concerns but does not necessarily correspond to measurable reductions in average crop productivity. However, previous studies in the Ayeyawady Region have identified salinity intrusion, flooding, and cyclone damage as major constraints to rice production [21,23]. Zaw and Charoenratana [57] revealed that climate change has a significant effect on crop production in the CDZ.

Among the agricultural resource constraint factors, water availability represents a critical constraint affecting food supply stability in semi-arid agricultural systems. This finding highlighted the importance of irrigation improvement and water-efficient practices. Soil fertility loss threatens long-term agricultural sustainability by reducing productivity potential and increasing dependence on external inputs [58]. Soil fertility decline was a factor influencing farmers’ decision-making in crop production [59].

### 4.3. Spatial Heterogeneity in Grain Self-Sufficiency

Grain self-sufficiency is important for supporting food security strategies at the national and regional levels. This study found that Ayeyawady and Bago serve as Myanmar’s staple crop-producing regions, creating surpluses that must flow via domestic market channels to food deficit zones. Similarly, Niu et al. [10,60] and Zhang et al. [61] found that rice is concentrated in south China, wheat in western China, maize in north and northwest China, and soybean in northeast China. The geographic clustering of crop production may increase systemic risk by concentrating national food production in a limited number of production centers. Disruptions in these production clusters could lead to food insecurity across the country.

The declining trend in Rakhine State is of particular concern. Rakhine has suffered severe political instability and population displacement since 2017, which has almost certainly contributed to farmland abandonment and production decline [62]. Addressing the declining capacity of staple food production may require political and agronomic intervention [63,64]. Similarly, Chin State’s continuing deterioration reflects agricultural resource constraints and climate-related risks.

## 5. Study Limitations

This study has some limitations. The key limitation is that the primary household survey was restricted to two districts in the CDZ (Yamethin and Ottara). As a result, the findings on the influencing factors may not fully represent the diversity of agricultural systems across the country. Moreover, the land-cover data derived from the GLC_FCS30D dataset have a reported global accuracy of about 80.88%. Misclassification is possible, especially in complex upland agro-forestry zones. Therefore, farmland area estimates for hilly states (Chin, Kachin, Shan) should be interpreted with this uncertainty in mind.

This study mainly assessed food availability via grain self-sufficiency. Food security is a multidimensional concept, including availability, access, utilization, and stability. States/regions with production surpluses may still experience household food insecurity resulting from poverty, unstable markets, and inadequate dietary diversification. Population estimates for recent years may be influenced by migration and displacement. Therefore, the grain self-sufficiency rate should be interpreted as an important component of food security rather than a comprehensive measure.

Finally, this study did not model the market and trade mechanisms behind inter-regional grain flows due to a lack of detailed information. The assumption that production surplus areas transfer grain to deficit regions is empirically reasonable, but this research does not directly measure it using trade or transport data. Future research should address these limitations through combining remote-sensing-based crop-type mapping validated with ground truth data; and supply chain analysis of inter-regional food flows using market survey, market price, and transport data.

## 6. Conclusions

This research studied changes in farmland area, cropping patterns, crop production, and grain self-sufficiency across Myanmar’s 14 states and regions from 2000 to 2025, revealing sustained farmland expansion and growing agricultural intensification. The results are as follows.

- While double cropping remained the primary system, the rapid expansion of multiple cropping represented structural intensification in response to both local demand and export opportunities.
- Agricultural production in the CDZ is supported by interacting environmental and socioeconomic factors, highlighting the need for climate-smart agricultural practices and improved market support mechanisms.
- Although Myanmar maintained overall grain self-sufficiency, geographical disparities in production indicate the strategic role of surplus-producing regions in supporting national food security.

The results imply that geographically targeted investment in irrigation, rural roads, and market infrastructure is urgently needed in grain deficit regions, particularly Chin State and Mandalay Region, to improve staple food sufficiency. Furthermore, targeted policies should be implemented to maintain a balance between staple food and commercial crop production in response to the rapid expansion of maize and pulse production in upland areas. The declining trend of agricultural capacity in Rakhine and Chin States should be tackled through agricultural restoration programs that address both the agronomic and political dimensions of farmland abandonment. This study provides a critical evidence base for farmers, policymakers, and development planners to improve sustainable farmland management, agricultural development planning, and long-term food security policy development in Myanmar.

## Figures and Tables

**Table 1:** Farmland area trends in Myanmar from 2000 to 2025 at the state/region level.

State/Region	2000 (Mha)	2025 (Mha)	Total Change	CAGR	M–K τ	*p*-Value	CV
Kayin	0.2992	0.5413	+80.92%	+2.40%	0.81	0.001	0.205
Kachin	0.2375	0.3792	+59.66%	+1.89%	0.61	0.001	0.178
Shan	0.9820	1.5043	+53.19%	+1.72%	0.93	0.001	0.134
Bago	1.8940	2.7250	+43.88%	+1.47%	0.57	0.001	0.092
Sagaing	2.2270	2.9981	+34.63%	+1.20%	0.86	0.001	0.106
Tanintharyi	0.2880	0.3871	+34.41%	+1.19%	0.40	0.004	0.109
Mon	0.4890	0.6295	+28.73%	+1.02%	0.32	0.021	0.112
Kayah	0.0742	0.0888	+19.68%	+0.72%	0.32	0.020	0.096
Ayeyawady	2.5960	3.0132	+16.07%	+0.60%	0.73	0.001	0.044
Yangon	0.7260	0.8303	+14.37%	+0.54%	0.34	0.015	0.063
Magway	1.8010	1.9741	+9.61%	+0.37%	−0.04	0.791	0.158
Mandalay	1.7240	1.7327	+0.50%	+0.02%	−0.15	0.289	0.124
Rakhine	0.5510	0.4440	−19.42%	−0.86%	−0.33	0.017	0.101
Chin	0.1098	0.0548	−50.09%	−2.74%	−0.66	0.001	0.281

CAGR = compound annual growth rate, M–K τ = Mann–Kendall tau (τ), CV = coefficient of variation.

**Table 2:** Area of cropping patterns in Myanmar from 2000 to 2025 (Mha).

Year	Double Cropping Area	Mixed Cropping Area
2000	3.7397	1.1918
2005	4.7951	1.8955
2010	5.7963	3.8554
2015	5.4292	2.3949
2020	4.7473	1.3605
2025	5.8286	1.5082
Mean	5.0560	2.0343
Standard deviation (SD)	0.7975	0.9905

**Table 3:** Changes in cropping pattern areas in Myanmar from 2000 to 2025 (Mha).

Crop Types/Years	2000	2005	2010	2015	2020	2025	SD	Total Change
Grains								
Rice	6.3588	7.3392	8.0472	7.2119	6.9618	7.1330	0.5482	+12.17%
Wheat	0.0801	0.1121	0.1016	0.0963	0.0563	0.0514	0.0249	−35.86%
Maize	0.2173	0.3209	0.3893	0.4719	0.5564	0.6277	0.1521	+188.83%
Oilseeds								
Groundnut	0.5900	0.7305	0.8774	0.9692	1.1404	1.3225	0.2674	+124.14%
Sesame	1.3387	1.2634	1.5200	1.5297	1.5374	1.6479	0.1429	+23.10%
Sunflower	0.4982	0.6900	0.8587	0.4460	0.2396	0.4330	0.2170	−13.08%
Pulses								
Black gram	0.6200	0.8150	1.0550	1.1331	0.9381	1.0538	0.1905	+69.97%
Green gram	0.7422	0.9490	1.1214	1.2100	1.1534	1.3104	0.2041	+76.55%
Soybean	0.1141	0.1558	0.1692	0.1485	0.1315	0.1485	0.0193	+30.14%
Pigeonpea	0.3622	0.5338	0.6333	0.6479	0.4318	0.4618	0.1141	+27.49%
Lablab bean	0.0886	0.0992	0.1153	0.1238	0.1020	0.1210	0.0139	+36.53%
Chickpea	0.1663	0.2238	0.3314	0.3723	0.3610	0.4140	0.0957	+148.91%
Others								
Potato	0.0291	0.0348	0.0389	0.0368	0.0304	0.0275	0.0046	−5.56%
Cotton	0.3088	0.3318	0.3509	0.2914	0.1574	0.2206	0.0737	−28.57%
Sugarcane	0.1388	0.1336	0.1514	0.1619	0.1773	0.1696	0.0173	+22.16%

**Table 4:** Changes in dominant crop areas in Myanmar from 2000 to 2025 (%).

		Grains		Pulses	Oilseeds	Others
State/Region	Rice	Wheat	Maize			
Kachin	+16.86	−58.23	+272.92	+37.19	+10.70	−58.44
Kayah	−4.24	−100.00	+250.45	−56.54	+59.97	−84.61
Kayin	+12.67	0.00	+1880.30	+240.86	+90.99	−63.16
Chin	−47.24	+443.15	−69.68	−64.63	+8.30	−48.58
Sagaing	+18.33	−42.97	+51.45	+47.72	+83.85	−44.15
Tanintharyi	−24.25	0.00	−97.06	+106.76	−33.59	−100.00
Bago	+19.17	0.00	+350.95	+105.61	+113.40	−57.41
Magway	+10.42	−77.43	−65.54	−3.16	+59.14	−16.15
Mandalay	−30.23	−82.66	−15.07	+23.19	+41.95	−45.89
Mon	−3.16	0.00	−62.96	+78.31	+39.19	−99.59
Rakhine	−10.39	0.00	0.00	−79.16	+7.67	−91.77
Yangon	+0.88	0.00	+1530.56	+49.29	−15.51	−87.12
Shan	+16.43	−29.23	+269.27	+10.84	+65.10	+99.83
Ayeyawady	+11.17	−100.00	+2.50	+31.97	+101.47	−99.83

Pulses = black gram, green gram, soybean, pigeonpea, lablab bean, and chickpea; oilseeds = sesame, sunflower, and groundnut; others = potato, cotton, and sugarcane.

**Table 5:** Changes in major crop production in Myanmar from 2000 to 2025 (million tons).

Crop Types/Years	2000	2005	2010	2015	2020	2025	SD	Total Change
Grains								
Rice	21.64	27.06	32.79	26.21	25.98	29.82	3.78	37.76%
Wheat	0.09	0.15	0.18	0.18	0.11	0.10	0.04	6.34%
Maize	0.38	0.93	1.37	1.75	2.07	2.43	0.75	546.51%
Oilseeds								
Groundnut	0.73	1.04	1.40	1.52	1.60	2.08	0.47	185.10%
Sesame	0.43	0.46	0.82	0.83	0.66	0.94	0.21	116.81%
Sunflower	0.48	0.84	1.32	0.66	0.21	0.35	0.40	−27.77%
Pulses								
Black gram	0.55	0.99	1.62	1.14	0.92	1.42	0.38	157.67%
Green gram	0.54	0.92	1.45	1.09	0.99	1.28	0.31	138.11%
Soybean	0.11	0.19	0.26	0.15	0.14	0.15	0.05	38.04%
Pigeonpea	0.32	0.60	0.82	0.60	0.32	0.35	0.20	9.53%
Lablab bean	0.06	0.09	0.13	0.16	0.12	0.15	0.04	140.22%
Chickpea	0.12	0.26	0.46	0.57	0.48	0.55	0.18	377.61%
Others								
Potato	0.31	0.47	0.57	0.56	0.47	0.43	0.10	37.60%
Cotton	0.16	0.33	0.54	0.51	0.26	0.42	0.15	168.67%
Sugarcane	6.17	7.26	9.35	10.14	11.55	11.54	2.22	86.87%

## Data Availability

The data presented in this study are available on request from the corresponding author. The data are not publicly available due to privacy restrictions.

## Supplementary Materials

The following supporting information can be downloaded at https://www.mdpi.com/article/10.3390/foods15173066/s1. Supplementary Material S1. Changes in farmland area by states/regions in Myanmar, 2000–2025. Supplementary Material S2. Changes in cropping pattern by states/regions in Myanmar, 2000–2025. Supplementary Material S3. Changes in crop production by states/regions in Myanmar, 2000–2025. Supplementary Material S4. Farmer questionnaire used for the household survey. Supplementary Material S5. Staple food self-sufficiency at the States/Regions level in Myanmar, 2000–2025.

## Appendix A

**Table A1:** Description and coding of dependent and independent variables.

Variable Category	Variable	Measurement/Coding
Dependent variables	Crop production	Continuous variable (ton ha^−1^)
	Farmland area	Continuous variable (ha)
Climate-related factors	Climate change	Binary variable (1 = yes, 0 = no)
	Drought	Binary variable (1 = yes, 0 = no)
	Floods	Binary variable (1 = yes, 0 = no)
	Erratic rainfall	Binary variable (1 = yes, 0 = no)
Agricultural resource constraint factors	Sufficient water availability	Binary variable (1 = sufficient water availability, 0 = insufficient water availability)
	Soil fertility loss	Binary variable (1 = soil fertility loss, 0 = no soil fertility loss)
	Limited access to good quality seed	Binary variable (1 = limited access to quality seed, 0 = sufficient access)
	Labor scarcity	Binary variable (1 = labor scarcity, 0 = no labor scarcity)
	Lack of market-based incentives	Binary variable (1 = lack of market incentives, 0 = sufficient incentives)

**Table A2:** Tests of MANOVA assumptions.

Assumption	Test	Statistic	*p*-Value	Interpretation
Homogeneity of covariance matrices	Box’s M test	Box’s M = 308.206	0.075	Satisfied
Equality of error variance	Levene’s test: Crop production	F = 1.094	0.226	Satisfied
Equality of error variance	Levene’s test: Farmland area	F = 1.299	0.014	Slight violation

Note: Although Levene’s test indicated heterogeneity of variance for farmland area (*p* < 0.05), Pillai’s Trace statistics were used because they are robust to moderate violations of variance–covariance assumptions, particularly when sample sizes are large.

**Table A3:** Multivariate effects of climate-related factors and agricultural resource constraint factors on farmland area and crop production.

Effect	Pillai’s Trace	*F*	Hypothesis *df*	Error *df*	*p*-Value	Partial *η*^2^
Climate change	0.005	1.568	2.000	589.000	0.209	0.005
Drought	0.012	3.445	2.000	589.000	0.033 *	0.012
Floods	0.003	0.962	2.000	589.000	0.383	0.003
Erratic rainfall	0.011	3.323	2.000	589.000	0.037 *	0.011
Sufficient water availability	0.037	11.191	2.000	589.000	<0.001 ***	0.037
Soil fertility loss	0.034	10.263	2.000	589.000	<0.001 ***	0.034
Limited access to good quality seed access	0.010	3.005	2.000	589.000	0.050	0.010
Labor scarcity	0.002	0.620	2.000	589.000	0.538	0.002
Lack of market-based incentives	0.001	0.339	2.000	589.000	0.712	0.001

Note: MANOVA was conducted using Pillai’s Trace statistics. Significant effects indicate that predictors influenced the combined dependent variables (crop production and farmland area). * *p* < 0.05; *** *p* < 0.001.

**Table A4:** Univariate effects of climate-related factors and agricultural resource constraint factors on farmland area and crop production.

	Independent Variables	Yes, N (Mean ± SD)	No, N (Mean ± SD)	*F*	*p*-Value	Partial *η*^2^
Crop production (ton/ha)	Climate change	455 (3.551 ± 0.937)	145 (3.735 ± 0.954)	3.134	0.077	0.005
	Drought	158 (3.763 ± 0.941)	442 (3.545 ± 0.939)	6.618	0.010 *	0.011
	Floods	508 (3.585 ± 0.937)	92 (3.651 ± 0.939)	0.290	0.590	<0.001
	Erratic rainfall	173 (3.674 ± 0.974)	427 (3.563 ± 0.920)	1.738	0.188	0.003
	Sufficient water availability	269 (3.752 ± 0.960)	331 (3.467 ± 0.898)	12.706	<0.001 *	0.021
	Soil fertility loss	335 (3.432 ± 0.889)	265 (3.801 ± 0.956)	20.502	<0.001 *	0.034
	Limited access to good quality seed	303 (3.511 ± 0.946)	297 (3.682 ± 0.920)	5.174	0.023 *	0.009
	Labor scarcity	476 (3.599 ± 0.926)	124 (3.581 ± 0.982)	0.135	0.713	<0.001
	Lack of market-based incentives	468 (3.595 ± 0.922)	132 (3.625 ± 0.989)	0.214	0.644	<0.001
	R^2^ = 0.085 (Adjusted R^2^ = 0.072)					
Farmland area (ha)	Climate change	455 (2.493 ± 1.236)	145 (2.521 ± 1.118)	0.007	0.931	<0.001
	Drought	158 (2.547 ± 1.322)	442 (2.483 ± 1.165)	0.279	0.597	<0.001
	Floods	508 (2.467 ± 1.157)	92 (2.681 ± 1.447)	1.635	0.202	0.003
	Erratic rainfall	173 (2.653 ± 1.381)	427 (2.438 ± 1.125)	4.921	0.027 *	0.008
	Sufficient water availability	269 (2.657 ± 1.271)	331 (2.372 ± 1.139)	9.686	0.002 *	0.016
	Soil fertility loss	335 (2.504 ± 1.145)	265 (2.494 ± 1.283)	0.062	0.804	<0.001
	Limited access to good quality seed	303 (2.451 ± 1.197)	297 (2.549 ± 1.310)	0.841	0.360	0.001
	Labor scarcity	476 (2.533 ± 1.249)	124 (2.372 ± 1.026)	1.106	0.293	0.002
	Lack of market-based incentives	468 (2.511 ± 1.233)	132 (2.460 ± 1.116)	0.465	0.496	0.001
	R^2^ = 0.031 (adjusted R^2^ = 0.016)					

Note: Values are presented as mean ± standard deviation (SD). Partial *η*^2^ represents the effect size. Statistical significance was determined at *p* < 0.05. * *p* < 0.05.

**Table A5:** Model summary.

Dependent Variable	*F*	*p*-Value	*R* ^2^	Adjusted *R*^2^
Crop production	6.126	<0.001	0.085	0.072
Farmland area	2.071	0.030	0.031	0.016
