# Supplementary material for: Farmland and Cropping Pattern Dynamics in Myanmar: Implications for Food Security and Sustainable Agriculture
Source: Foods. 2026 Aug 29;15(17):3066. doi: 10.3390/foods15173066 (PMC13564485; doi:10.3390/foods15173066)
Supplement: Supplementary file 1 [file foods-15-03066-s001.zip › Supplementary Material S1.pdf]

Supplementary S1

Table S1. Changes in farmlands area by states/regions in Myanmar, 2000-2025 (ha)

Year	Kachin	Kayah	Kayin	Chin	Sagaing	Tanintharyi	Bago	Magway	Mandalay	Mon	Rakhine	Yangon	Shan	Ayeyarwady	Total area (ha)
2000	237500	74200	299200	109800	2227000	288000	1894000	1801000	1724000	489000	551000	726000	982000	2596000	13998700
2001	238200	74400	300000	110100	2233000	288800	1899000	1806000	1729000	490300	552500	728000	985000	2603000	14037300
2002	239100	74700	301200	110500	2242000	290000	1907000	1814000	1736000	492300	554800	731100	989000	2613000	14094700
2003	239300	74800	301500	110600	2244000	290300	1909000	1816000	1738000	492800	555400	731900	990000	2615000	14108600
2004	236694	73986	298267	109505	2220342	287096	1888505	1796453	1719890	487587	549319	723659	979137	2588958	13959398
2005	248606	83097	342649	118421	2315224	324220	1993863	1808181	1816542	576875	590201	747478	1051546	2721000	14737903
2006	303025	85291	362559	124790	2531864	362864	2169796	1935976	1845229	663891	648005	766188	1141183	2783982	15724643
2007	344080	88235	390092	131851	2603547	388687	2236840	1941759	2671621	691466	663327	772726	1197466	2882139	17003836
2008	369055	91372	417353	134332	2620234	392591	2262856	2492552	1884488	695694	676884	629391	1231323	2874342	16772467
2009	396383	92623	432784	134607	2688022	386347	2321418	2509994	1865553	707250	679079	632910	1256478	2898174	17001622
2010	399333	95845	445696	135322	2695875	384731	2315903	2640186	1889471	714894	682281	633477	1286967	2904386	17224367
2011	358753	94814	452462	105667	2696768	380811	2195507	2654331	1653989	663836	620645	765912	1297596	2759672	16700763
2012	310257	92637	501846	97264	2603751	376402	2235750	2076991	1506331	625780	606738	766697	1283142	2744087	15827673
2013	307587	92547	516289	93365	2741737	372927	2250220	2274871	1605708	592465	606286	765295	1290540	2777715	16287552
2014	312194	95679	532241	94179	2691342	379232	2256971	2089031	1515286	597426	604369	769478	1286388	2824383	16048199
2015	332438	100000	535058	87899	2928727	385560	2269045	2112352	1583324	600838	606629	769412	1319336	2862956	16493574
2016	332343	100000	535089	87899	2928669	385554	2268994	2120557	1583272	600832	604855	771125	1319281	2862515	16500985
2017	335121	102110	512628	80338	2938636	310035	2265488	2058760	1608924	606371	602422	738939	1335048	2868099	16362919
2018	344137	93599	500921	77172	2917932	385235	2273106	1884519	1676361	603708	569818	755620	1349016	2891159	16322303
2019	357691	94020	505445	74948	2897616	385206	2261307	1556572	1622294	604603	535195	757016	1366609	2895152	15913674
2020	403013	92420	507185	66134	2950719	383095	2232940	1571640	1623116	605630	515929	743507	1414088	2915896	16025312
2021	403131	92420	507185	66134	2951581	383095	2232940	1571640	1623116	605630	515929	743507	1414088	2915896	16026292
2022	398234	80583	538740	58039	3045047	383653	2253875	1597470	1643630	608069	523375	746634	1449282	2907686	16234317
2023	394084	83999	537476	46948	2993749	385137	2349257	1684246	1690530	616442	525630	747650	1461221	2963351	16479720
2024	401452	84684	539412	53300	2993737	385511	2622295	1884352	1733921	623485	523237	823235	1488963	2982239	17139823
2025	379264	88807	541324	54803	2998165	387100	2725060	1974131	1732761	629529	444050	830273	1504359	3013198	17302824

Table S2. Farmlands area in Myanmar at national level (ha)

Year	Farmland area (GIS data)	Farmland area (Official data)
2000	13943122	13998700
2001	13981843	14037300
2002	14031738	14094700
2003	14040512	14108600
2004	13978160	13959398
2005	13998400	14737903
2006	14073853	15724643
2007	14136904	17003836
2008	14277314	16772467
2009	14358750	17001622
2010	14362849	17224367
2011	14381648	16700763
2012	14457031	15827673
2013	14571684	16287552
2014	14596098	16048199
2015	14608718	16493574
2016	14698783	16500985
2017	14791949	16362919
2018	14916614	16322303
2019	15010824	15913674
2020	15102335	16025312
2021	15162957	16026292
2022	15142096	16234317
2023		16479720
2024		17139823
2025		17302824

Google earth engine code:

https://code.earthengine.google.com/e9239de7a6aa8cffdd359090e62b3510
