# Supplementary material for: Farmland and Cropping Pattern Dynamics in Myanmar: Implications for Food Security and Sustainable Agriculture
Source: Foods. 2026 Aug 29;15(17):3066. doi: 10.3390/foods15173066 (PMC13564485; doi:10.3390/foods15173066)
Supplement: Supplementary file 1 [file foods-15-03066-s001.zip › Supplementary Material S2.pdf]

Supplementary S2

Table S3. Changes in cropping pattern by Kachin State between 2000 and 2025

No.	Crop types	2000 Area (1000*ha)	2025 Area (1000*ha)	Change in %	2010 Area (1000*ha)	2025 Area (1000*ha)	Change in %
1	Rice	166.26	194.29	16.86	267.13	194.29	-27.27
2	Wheat	0.03	0.01	-58.23	0.09	0.01	-85.78
3	Maize	5.94	22.13	272.92	13.48	22.13	64.23
4	Groundnut	18.94	13.34	-29.58	25.61	13.34	-47.93
5	Sesame	4.59	8.06	75.72	10.03	8.06	-19.69
6	Sunflower	0.00	4.65	*	0.00	4.65	*
7	Black gram	0.05	0.00	-99.17	0.10	0.00	-99.61
8	Green gram	0.65	0.41	-36.65	1.04	0.41	-60.40
9	Soybean	5.41	8.47	56.61	7.48	8.47	13.23
10	Pigeon pea	0.45	0.12	-73.62	0.42	0.12	-71.18
11	Lablab bean	0.00	0.00	0.00	0.00	0.00	N/A
12	Chickpea	0.00	0.00	0.00	0.00	0.00	N/A
13	Potato	2.32	1.47	-36.60	3.03	1.47	-51.34
14	Cotton	0.00	0.00	-100.00	0.01	0.00	-100.00
15	Sugarcane	7.88	2.77	-64.86	5.38	2.77	-48.56

* No baseline area

Table S4. Changes in cropping pattern by Kayah State between 2000 and 2025

No.	Crop types	2000 Area (1000*ha)	2025 Area (1000*ha)	Change in %	2010 Area (1000*ha)	2025 Area (1000*ha)	Change in %
1	Rice	31.78	30.43	-4.24	45.46	30.43	-33.05
2	Wheat	0.07	0.00	-100.00	0.01	0.00	-100.00
3	Maize	6.33	22.20	250.45	8.40	22.20	164.42
4	Groundnut	5.33	2.66	-50.05	6.69	2.66	-60.17
5	Sesame	3.70	9.92	167.81	7.25	9.92	36.86
6	Sunflower	0.00	1.87	*	0.00	1.87	*
7	Black gram	0.00	0.13	*	0.00	0.13	*
8	Green gram	0.63	0.41	-35.20	1.21	0.41	-66.30
9	Soybean	3.34	0.51	-84.68	4.44	0.51	-88.46
10	Pigeon pea	3.99	2.37	-40.64	4.09	2.37	-42.10
11	Lablab bean	0.04	0.08	96.23	0.12	0.08	-30.67
12	Chickpea	0.21	0.07	-67.88	0.20	0.07	-66.40
13	Potato	0.39	0.07	-82.88	0.65	0.07	-89.82
14	Cotton	0.04	0.00	-100.00	0.04	0.00	-100.00
15	Sugarcane	0.00	0.00	0.00	0.00	0.00	0.00

* No baseline area

Table S5. Changes in cropping pattern by Kayin State between 2000 and 2025

No.	Crop types	2000 Area (1000*ha)	2025 Area (1000*ha)	Change in %	2010 Area (1000*ha)	2025 Area (1000*ha)	Change in %
1	Rice	194.33	218.95	12.67	274.85	218.95	-20.34
2	Wheat	0.00	0.00	0.00	0.00	0.00	0.00
3	Maize	1.06	21.08	1880.30	15.74	21.08	33.90
4	Groundnut	10.07	14.73	46.37	13.23	14.73	11.36
5	Sesame	8.00	18.77	134.58	14.76	18.77	27.14
6	Sunflower	0.00	1.00	*	0.00	1.00	*
7	Black gram	0.03	0.88	3261.54	0.50	0.88	76.49
8	Green gram	4.75	27.87	486.12	20.43	27.87	36.43
9	Soybean	3.76	5.10	35.67	3.68	5.10	38.59
10	Pigeon pea	0.01	0.17	1209.09	0.28	0.17	-36.47
11	Lablab bean	2.06	2.16	4.80	2.23	2.16	-2.91
12	Chickpea	0.00	0.00	0.00	0.00	0.00	0.00
13	Potato	0.00	0.00	0.00	0.00	0.00	0.00

14	Cotton	0.00	0.00	0.00	0.00	0.00	0.00
15	Sugarcane	6.47	2.38	-63.16	3.13	2.38	-23.80

* No baseline area

Table S6. Changes in cropping pattern by Chin State between 2000 and 2025

No.	Crop types	2000 Area (1000*ha)	2025 Area (1000*ha)	Change in %	2010 Area (1000*ha)	2025 Area (1000*ha)	Change in %
1	Rice	44.22	23.33	-47.24	56.23	23.33	-58.51
2	Wheat	0.08	0.43	443.15	0.08	0.43	421.95
3	Maize	34.07	10.33	-69.68	37.12	10.33	-72.17
4	Groundnut	1.12	1.30	15.95	1.69	1.30	-22.74
5	Sesame	2.61	1.79	-31.32	3.09	1.79	-42.02
6	Sunflower	0.00	0.95	*	0.00	0.95	*
7	Black gram	0.01	0.01	9.38	0.01	0.01	133.33
8	Green gram	0.10	0.11	3.16	0.24	0.11	-55.99
9	Soybean	1.07	0.19	-82.01	1.35	0.19	-85.72
10	Pigeon pea	1.19	0.21	-82.10	1.17	0.21	-81.90
11	Lablab bean	0.06	0.26	324.34	0.21	0.26	24.04
12	Chickpea	0.00	0.07	*	0.00	0.07	*
13	Potato	1.42	1.10	-22.70	1.98	1.10	-44.81
14	Cotton	0.59	0.02	-96.44	0.49	0.02	-95.72
15	Sugarcane	0.36	0.10	-71.91	0.37	0.10	-72.91

* No baseline area

Table S7. Changes in cropping pattern by Sagaing Region between 2000 and 2025

No.	Crop types	2000 Area (1000*ha)	2025 Area (1000*ha)	Change in %	2010 Area (1000*ha)	2025 Area (1000*ha)	Change in %
1	Rice	693.38	820.50	18.33	927.73	820.50	-11.56
2	Wheat	68.74	39.20	-42.97	72.28	39.20	-45.77
3	Maize	50.89	77.08	51.45	79.59	77.08	-3.16
4	Groundnut	179.64	384.50	114.04	274.07	384.50	40.29
5	Sesame	346.00	430.60	24.45	364.18	430.60	18.24
6	Sunflower	0.00	151.30	*	0.00	151.30	*
7	Black gram	42.11	72.93	73.17	65.43	72.93	11.45
8	Green gram	148.76	228.27	53.45	214.28	228.27	6.53
9	Soybean	11.20	15.00	34.01	13.08	15.00	14.71
10	Pigeon pea	151.87	188.78	24.31	220.73	188.78	-14.48
11	Lablab bean	11.06	26.68	141.12	20.79	26.68	28.34
12	Chickpea	110.28	170.42	54.53	151.53	170.42	12.46
13	Potato	2.27	2.65	16.60	3.00	2.65	-11.71
14	Cotton	49.59	32.05	-35.38	56.96	32.05	-43.74
15	Sugarcane	100.28	50.28	-49.86	64.91	50.28	-22.54

* No baseline area

Table S8. Changes in cropping pattern by Tanintharyi Region between 2000 and 2025

No.	Crop types	2000 Area (1000*ha)	2025 Area (1000*ha)	Change in %	2010 Area (1000*ha)	2025 Area (1000*ha)	Change in %
1	Rice	123.12	93.27	-24.25	150.44	93.27	-38.01
2	Wheat	0.00	0.00	0.00	0.00	0.00	0.00
3	Maize	0.06	0.00	-97.06	0.08	0.00	-98.10
4	Groundnut	0.22	0.00	-98.52	0.03	0.00	-88.41
5	Sesame	0.46	0.38	-17.22	0.67	0.38	-42.78
6	Sunflower	0.00	0.07	*	0.00	0.07	*
7	Black gram	0.15	0.82	449.73	0.12	0.82	575.75
8	Green gram	0.06	0.05	-23.33	0.07	0.05	-29.88
9	Soybean	0.00	0.00	0.00	0.00	0.00	0.00
10	Pigeon pea	0.21	0.01	-95.85	0.03	0.01	-69.44
11	Lablab bean	0.00	0.00	0.00	0.00	0.00	0.00

12	Chickpea	0.00	0.00	0.00	0.00	0.00	0.00
13	Potato	0.00	0.00	0.00	0.00	0.00	0.00
14	Cotton	0.00	0.00	0.00	0.00	0.00	0.00
15	Sugarcane	0.63	0.00	-100.00	0.00	0.00	0.00

* No baseline area

Table S9. Changes in cropping pattern by Bago Region between 2000 and 2025

No.	Crop types	2000 Area (1000*ha)	2025 Area (1000*ha)	Change in %	2010 Area (1000*ha)	2025 Area (1000*ha)	Change in %
1	Rice	1087.09	1295.46	19.17	1389.85	1295.46	-6.79
2	Wheat	0.00	0.00	0.00	0.00	0.00	0.00
3	Maize	2.10	9.46	350.95	0.77	9.46	1125.85
4	Groundnut	54.15	119.69	121.03	76.94	119.69	55.56
5	Sesame	56.37	96.50	71.19	75.89	96.50	27.16
6	Sunflower	0.00	19.66	*	0.00	19.66	*
7	Black gram	305.84	446.73	46.07	436.60	446.73	2.32
8	Green gram	111.51	401.27	259.84	162.39	401.27	147.10
9	Soybean	10.79	21.53	99.46	15.56	21.53	38.40
10	Pigeon pea	5.91	8.77	48.28	6.87	8.77	27.72
11	Lablab bean	9.80	16.22	65.51	11.99	16.22	35.31
12	Chickpea	4.12	26.58	545.35	10.48	26.58	153.62
13	Potato	0.21	0.01	-94.69	0.25	0.01	-95.65
14	Cotton	13.88	6.27	-54.82	11.43	6.27	-45.14
15	Sugarcane	52.21	21.96	-57.95	18.75	21.96	17.13

* No baseline area

Table S10. Changes in cropping pattern by Magway Region between 2000 and 2025

No.	Crop types	2000 Area (1000*ha)	2025 Area (1000*ha)	Change in %	2010 Area (1000*ha)	2025 Area (1000*ha)	Change in %
1	Rice	244.73	270.23	10.42	426.34	270.23	-36.62
2	Wheat	0.57	0.13	-77.43	0.61	0.13	-78.82
3	Maize	33.93	11.69	-65.54	36.98	11.69	-68.38
4	Groundnut	150.94	372.47	146.76	160.36	372.47	132.27
5	Sesame	499.93	568.49	13.71	538.19	568.49	5.63
6	Sunflower	0.00	94.83	*	0.00	94.83	*
7	Black gram	5.94	24.71	315.77	12.15	24.71	103.40
8	Green gram	247.86	224.29	-9.51	279.23	224.29	-19.68
9	Soybean	4.65	1.25	-73.13	5.09	1.25	-75.48
10	Pigeon pea	152.17	82.74	-45.62	174.07	82.74	-52.47
11	Lablab bean	49.52	48.99	-1.07	55.64	48.99	-11.95
12	Chickpea	54.84	116.70	112.82	74.47	116.70	56.72
13	Potato	1.77	0.31	-82.62	1.94	0.31	-84.16
14	Cotton	111.08	100.13	-9.86	140.01	100.13	-28.48
15	Sugarcane	12.58	4.74	-62.34	5.76	4.74	-17.72

* No baseline area

Table S11. Changes in cropping pattern by Mandalay Region between 2000 and 2025

No.	Crop types	2000 Area (1000*ha)	2025 Area (1000*ha)	Change in %	2010 Area (1000*ha)	2025 Area (1000*ha)	Change in %
1	Rice	323.93	225.99	-30.23	390.35	225.99	-42.11
2	Wheat	11.25	1.95	-82.66	11.39	1.95	-82.87
3	Maize	19.10	16.22	-15.07	19.29	16.22	-15.90
4	Groundnut	117.56	258.44	119.85	150.05	258.44	72.24
5	Sesame	432.84	447.50	3.39	464.78	447.50	-3.72
6	Sunflower	0.00	75.35	*	0.00	75.35	*
7	Black gram	12.58	13.01	3.45	30.05	13.01	-56.70
8	Green gram	70.59	110.88	57.08	163.50	110.88	-32.18
9	Soybean	13.80	15.02	8.84	12.61	15.02	19.07
10	Pigeon pea	178.98	153.81	-14.06	195.23	153.81	-21.21

11	Lablab bean	15.31	14.35	-6.25	17.16	14.35	-16.39
12	Chickpea	36.11	96.21	166.43	92.15	96.21	4.41
13	Potato	1.25	0.86	-31.49	1.67	0.86	-48.70
14	Cotton	116.21	80.06	-31.11	140.54	80.06	-43.03
15	Sugarcane	47.01	8.08	-82.82	16.70	8.08	-51.66

* No baseline area

Table S12. Changes in cropping pattern by Mon State between 2000 and 2025

No.	Crop types	2000	2025	Change in %	2010	2025	Change in %
		Area (1000*ha)	Area (1000*ha)		Area (1000*ha)	Area (1000*ha)	
1	Rice	313.50	303.60	-3.16	407.58	303.60	-25.51
2	Wheat	0.00	0.00	0.00	0.00	0.00	*
3	Maize	0.02	0.01	-62.96	0.00	0.01	*
4	Groundnut	9.00	9.18	2.10	12.69	9.18	-27.65
5	Sesame	2.16	0.90	-58.57	6.71	0.90	-86.65
6	Sunflower	0.00	5.45	*	0.00	5.45	*
7	Black gram	3.52	3.82	8.49	5.15	3.82	-25.96
8	Green gram	8.83	21.05	138.45	12.48	21.05	68.72
9	Soybean	1.63	0.22	-86.57	1.82	0.22	-88.00
10	Pigeon pea	0.09	0.00	-100.00	0.10	0.00	-100.00
11	Lablab bean	0.00	0.00	0.00	0.00	0.00	0.00
12	Chickpea	0.00	0.00	0.00	0.00	0.00	0.00
13	Potato	0.00	0.00	0.00	0.00	0.00	0.00
14	Cotton	0.00	0.00	0.00	0.00	0.00	0.00
15	Sugarcane	7.17	0.03	-99.59	1.56	0.03	-98.13

* No baseline area

Table S13. Changes in cropping pattern by Rakhine State between 2000 and 2025

No.	Crop types	2000	2025	Change in %	2010	2025	Change in %
		Area (1000*ha)	Area (1000*ha)		Area (1000*ha)	Area (1000*ha)	
1	Rice	394.06	353.14	-10.39	500.80	353.14	-29.49
2	Wheat	0.00	0.00	0.00	0.00	0.00	0.00
3	Maize	0.00	0.01	*	0.07	0.01	-79.75
4	Groundnut	23.84	25.11	5.33	40.64	25.11	-38.21
5	Sesame	1.17	1.33	13.68	2.00	1.33	-33.52
6	Sunflower	0.00	0.49	*	0.00	0.49	*
7	Black gram	21.39	4.21	-80.32	25.53	4.21	-83.51
8	Green gram	1.36	0.47	-65.48	2.61	0.47	-81.99
9	Soybean	0.00	0.00	0.00	0.00	0.00	0.00
10	Pigeon pea	1.24	0.19	-85.00	1.42	0.19	-86.92
11	Lablab bean	0.00	0.01	*	0.01	0.01	-14.29
12	Chickpea	0.00	0.00	0.00	0.00	0.00	0.00
13	Potato	3.66	0.32	-91.16	3.80	0.32	-91.49
14	Cotton	0.00	0.00	0.00	0.00	0.00	0.00
15	Sugarcane	1.61	0.11	-93.15	1.00	0.11	-88.91

* No baseline area

Table S14. Changes in cropping pattern by Yangon Region between 2000 and 2025

No.	Crop types	2000	2025	Change in %	2010	2025	Change in %
		Area (1000*ha)	Area (1000*ha)		Area (1000*ha)	Area (1000*ha)	
1	Rice	566.21	571.19	0.88	563.83	571.19	1.31
2	Wheat	0.00	0.00	0.00	0.00	0.00	0.00
3	Maize	0.03	0.48	1530.56	0.04	0.48	1074.00
4	Groundnut	4.75	2.39	-49.65	4.32	2.39	-44.60
5	Sesame	2.20	1.66	-24.63	1.96	1.66	-15.49
6	Sunflower	0.00	1.82	*	0.00	1.82	*
7	Black gram	15.74	25.62	62.79	19.69	25.62	30.16
8	Green gram	116.19	171.25	47.39	145.51	171.25	17.69
9	Soybean	0.00	0.08	*	0.00	0.08	*

10	Pigeon pea	0.00	0.00	0.00	0.00	0.00	0.00
11	Lablab bean	0.00	0.00	0.00	0.00	0.00	0.00
12	Chickpea	0.00	0.00	0.00	0.00	0.00	0.00
13	Potato	0.00	0.00	0.00	0.00	0.00	0.00
14	Cotton	0.00	0.00	0.00	0.00	0.00	0.00
15	Sugarcane	10.63	1.37	-87.12	2.12	1.37	-35.46

* No baseline area

Table S15. Changes in cropping pattern by Shan State between 2000 and 2025

No.	Crop types	2000 Area (1000*ha)	2025 Area (1000*ha)	Change in %	2010 Area (1000*ha)	2025 Area (1000*ha)	Change in %
1	Rice	448.42	522.12	16.43	623.95	522.12	-16.32
2	Wheat	13.48	9.54	-29.23	16.17	9.54	-40.98
3	Maize	111.61	412.16	269.27	172.04	412.16	139.57
4	Groundnut	36.44	49.89	36.89	57.47	49.89	-13.20
5	Sesame	12.61	15.61	23.76	18.61	15.61	-16.12
6	Sunflower	0.00	15.49	*	0.00	15.49	*
7	Black gram	0.02	0.29	1288.24	0.12	0.29	147.55
8	Green gram	0.33	5.81	1643.08	1.06	5.81	448.21
9	Soybean	64.47	69.84	8.33	87.62	69.84	-20.29
10	Pigeon pea	26.46	23.68	-10.53	27.20	23.68	-12.94
11	Lablab bean	0.50	1.99	300.00	1.64	1.99	21.26
12	Chickpea	0.66	0.86	29.90	0.72	0.86	19.98
13	Potato	19.39	20.69	6.71	22.36	20.69	-7.47
14	Cotton	0.60	0.18	-69.42	1.01	0.18	-81.92
15	Sugarcane	27.24	73.51	169.82	31.68	73.51	132.05

* No baseline area

Table S16. Changes in cropping pattern by Ayeyawady Region between 2000 and 2025

No.	Crop types	2000 Area (1000*ha)	2025 Area (1000*ha)	Change in %	2010 Area (1000*ha)	2025 Area (1000*ha)	Change in %
1	Rice	1911.78	2125.36	11.17	2022.57	2125.36	5.08
2	Wheat	0.92	0.00	-100.00	0.84	0.00	-100.00
3	Maize	19.32	19.80	2.50	5.83	19.80	239.64
4	Groundnut	42.53	37.58	-11.64	53.33	37.58	-29.53
5	Sesame	9.16	9.36	2.17	11.82	9.36	-20.80
6	Sunflower	0.00	57.20	*	0.00	57.20	*
7	Black gram	324.90	420.20	29.33	459.43	420.20	-8.54
8	Green gram	58.16	97.04	66.84	117.14	97.04	-17.16
9	Soybean	15.28	11.14	-27.11	16.26	11.14	-31.49
10	Pigeon pea	1.83	0.81	-55.77	1.73	0.81	-53.29
11	Lablab bean	5.10	4.49	-11.85	5.61	4.49	-19.87
12	Chickpea	1.05	2.55	142.07	1.98	2.55	28.43
13	Potato	0.01	0.00	-100.00	0.01	0.00	-100.00
14	Cotton	0.31	0.00	-100.00	0.21	0.00	-100.00
15	Sugarcane	0.87	0.00	-99.77	0.16	0.00	-98.71

* No baseline area

Table S17. Changes in cropping pattern by Nay Pyi Taw between 2015 and 2025

No.	Crop types	2015 Area (1000*ha)	2025 Area (1000*ha)	Change in %	2020 Area (1000*ha)	2025 Area (1000*ha)	Change in %
1	Rice	71.66	85.28	19.00	75.34	85.28	13.18
2	Wheat	0.00	0.00	0.00	0.00	0.00	0.00
3	Maize	7.08	5.21	-26.51	6.00	5.21	-13.28
4	Groundnut	19.31	31.02	60.63	20.90	31.02	48.44
5	Sesame	30.07	26.52	-11.81	35.56	26.52	-25.44
6	Sunflower	0.00	3.13	*	0.46	3.13	584.41
7	Black gram	33.36	40.33	20.89	25.85	40.33	56.02
8	Green gram	28.98	21.12	-27.11	18.19	21.12	16.11

9	Soybean	0.23	0.07	-70.98	0.19	0.07	-63.99
10	Pigeon pea	0.33	0.33	-0.73	0.29	0.33	14.03
11	Lablab bean	6.59	5.71	-13.35	8.42	5.71	-32.17
12	Chickpea	1.28	0.85	-33.89	1.22	0.85	-30.53
13	Potato	0.68	0.00	-100.00	0.44	0.00	-100.00
14	Cotton	3.65	1.99	-45.36	3.79	1.99	-47.50
15	Sugarcane	4.19	4.36	4.06	4.72	4.36	-7.63

* No baseline area
