# Supplementary material for: Farmland and Cropping Pattern Dynamics in Myanmar: Implications for Food Security and Sustainable Agriculture
Source: Foods. 2026 Aug 29;15(17):3066. doi: 10.3390/foods15173066 (PMC13564485; doi:10.3390/foods15173066)
Supplement: Supplementary file 1 [file foods-15-03066-s001.zip › Supplementary Material S3.pdf]

Supplementary S3

Table S18. Trend of dominant/main crops production in Myanmar, 2000-2025 (ton)

Crop Types/Years	2000	2005	2010	2015	2020	2025
Rice	21644586	27057121	32786792	26210319	25982696	29817276
Wheat	90555	153833	180392	179307	105457	96295
Maize	375218	930084	1368362	1748547	2072482	2425802
Groundnut	730195	1040302	1395236	1517934	1595824	2081796
Sesamum	431951	461842	822544	827431	657993	936495
Sunflower	482740	835481	1317103	658552	210394	348706
Black gram	550519	986897	1618138	1142002	921783	1418531
Green gram	539216	919274	1448363	1090005	993965	1283902
Soybean	110548	188657	259448	154348	135132	152603
Pigeon pea	321615	603245	818004	602303	320313	352272
Lablab bean	61706	92042	133836	156909	115417	148232
Chickpea	115804	259689	461524	571458	481985	553091
Potato	311630	471189	565786	560306	468098	428794
Cotton	157451	332813	544494	512816	262277	423030
Sugarcane	6174000	7260000	9350000	10142383	11551111	11537093

Table S19. Changes in crop production by states/regions in Myanmar, 2000-2025

States & Region	Class	Change %
Kachin	Stable	4.24
Kayah	Significant increase	51.91
Kayin	Moderate increase	27.85
Chin	Significant decline	-63.49
Sagaing	Stable	13.84
Tanintharyi	Moderate decline	-22.58
Bago	Significant increase	47.63
Magway	Significant increase	37.57
Mandalay	Significant decline	-37.17
Mon	Moderate decline	-24.11
Rakhine	Moderate decline	-22.78
Yangon	Stable	-3.93
Shan	Significant increase	172.54
Ayeyarwady	Significant increase	55.79

Table S20. Factors influencing changes in farmland and crop production in Myanmar

Influencing factors	Percentage
Climate change (< 50%)	75.83
Drought	26.33
Floods	84.67
Erratic rainfall	28.83
Sufficient water availability	44.83
Soil fertility loss	55.83

Limited access to good quality seed	50.53
Labor scarcity	79.33
Lack of market-based incentives	78.00
