# Supplementary material for: Farmland and Cropping Pattern Dynamics in Myanmar: Implications for Food Security and Sustainable Agriculture
Source: Foods. 2026 Aug 29;15(17):3066. doi: 10.3390/foods15173066 (PMC13564485; doi:10.3390/foods15173066)
Supplement: Supplementary file 1 [file foods-15-03066-s001.zip › Supplementary Material S4.pdf]

Supplementary S4

Farmer Questionnaire

The following farmer's survey questionnaire is designed to assess Farmland and Cropping Pattern Dynamics in Myanmar: Implications for Food Security and Sustainable Agriculture.

These research questions are dedicated to Mr. Saw Yan Naing's doctoral thesis who is studying at the University of Chinese Academy of Sciences, China. We would appreciate it if you could help us with our research for 30 to 45 min. We will never give your personal information to any parties and will only use it for research purposes.

1. Household Characteristics

Q.1 Household identification code ……………………………

Q.2 What is the gender? Male…… or female………………………..

Q.3 What is the age of the household head (years)? ……………………..

Q.4 Education (years completed)? ……………………………………

Q.5 Farming experience (years)? …………………………………….

Q.6 Family size? ……………………………………………………

Q.7 How many families member are in the labor force? ………………

2. Landholding information (to know area changes and corresponding crop pattern changes)

		2020		2025	
**Irrigated**	Q.1 Total area (acre)				
	Q.2 Crop name, area (acre) and yield per acre	1.<br>2.<br>3.	4.<br>5.<br>6.	1.<br>2.<br>3.	4.<br>5.<br>6.
**Rainfed**	Q.3 Total area (acre)				
	Q.4 Crop name, area (acre) and yield per acre	1.<br>2.<br>3.	4.<br>5.<br>6.	1.<br>2.<br>3.	4.<br>5.<br>6.

Q.1 What are the planting/harvesting dates of each crop?

C.1………./………..

C.2………./………..C.3………./………..C.4………./………..C.5………./………..C.6………./………..

Q.2 What are the reasons for changing cropping patterns?

1…………………...

2…………………...

3…………………...

4…………………...

5…………………...

3. To know the factors influencing changes in farmland and crop production

Q.1. Have you observed any of the following climate change events?

(a) Increase in temperature (summer season) ……

(b) Increase in temperature (rainy season) ………

(c) Decrease in rainfall ……………………………

(d) Drought ……………………………………….

(e) Floods …………………………………………

(f) Erratic rainfall …………………………………

(g) Storms wind …………………………………..

(h) Other (specify) ……………………………….

Q.2 Does climate change affect your primary crop production (<50%)? Yes……/No……

Q.3 What are the main sources of irrigation water?

(a) Dam …..

(b) Tubewell (government) …..

(c) Tubewell (private) …..

(d) Streams and ponds …..

(e) Rainfall …..

Q.3.1 Do you have sufficient water availability? Yes……/No……

Q.3.2 Do you currently satisfy water infrastructure? Yes……/No……

Q.3.3 Do you agree that it is important to use water much more efficiently? Yes……/No……

Q.4 How do you think about your agricultural soil?

(a) Increased soil erosion? Yes……/No……

(b) Loss of soil fertility? Yes……/No……

(c) Increased soil moisture deficit? Yes……/No……

Q.5 Constraints to crop production and marketing

(a) Limited access to quality inputs? Yes……/No……

(b) Limited access to good quality seed? Yes……/No……

(c) Pest and disease infestation? Yes……/No……

(d) Labor scarcity? Yes……/No……

(e) Lack of market-based incentives? Yes……/No……

(f) High transportation costs? Yes……/No……

Thank you very much for your time and empathy
