# Supplementary material for: Farmland and Cropping Pattern Dynamics in Myanmar: Implications for Food Security and Sustainable Agriculture
Source: Foods. 2026 Aug 29;15(17):3066. doi: 10.3390/foods15173066 (PMC13564485; doi:10.3390/foods15173066)
Supplement: Supplementary file 1 [file foods-15-03066-s001.zip › Supplementary Material S5.pdf]

Supplementary S5

Table S21. Estimated Urban population by States and Regions of Myanmar (million)

	2000	2005	2010	2015	2020	2025
Union	14.61	16.83	18.34	14.88	16.29	15.68
Kachin State	0.32	0.41	0.45	0.59	0.65	0.77
Kayah State	0.09	0.11	0.12	0.07	0.08	0.09
Kayin State	0.19	0.24	0.28	0.33	0.36	0.34
Chin State	0.09	0.11	0.12	0.10	0.11	0.08
Sagaing Region	0.97	1.13	1.24	0.91	1.00	1.09
Tanintharyi Region	0.40	0.47	0.53	0.34	0.37	0.37
Bago Region	1.25	1.39	1.49	1.07	1.17	1.13
Magway Region	0.87	0.98	1.06	0.59	0.64	0.68
Mandalay Region	2.25	2.60	2.85	2.14	2.35	2.12
Mon State	0.87	1.00	1.09	0.57	0.63	0.64
Rakhine State	0.53	0.61	0.66	0.35	0.39	0.47
Yangon Region	4.37	4.97	5.44	5.16	5.65	5.01
Shan State	1.14	1.38	1.50	1.40	1.53	1.62
Ayeyawady Region	1.27	1.45	1.52	0.87	0.96	0.89

Table S22. Estimated Rural population by States and Regions of Myanmar (million)

	2000	2005	2010	2015	2020	2025
Union	35.52	38.57	41.44	35.40	38.29	35.69
Kachin State	0.98	1.05	1.13	1.05	1.27	1.39
Kayah State	0.19	0.21	0.24	0.21	0.25	0.21
Kayin State	1.32	1.44	1.54	1.18	1.25	1.16
Chin State	0.39	0.41	0.43	0.38	0.41	0.29
Sagaing Region	4.45	4.90	5.31	4.41	4.59	4.89
Tanintharyi Region	0.99	1.10	1.19	1.07	1.12	1.03
Bago Region	3.89	4.22	4.52	3.80	3.77	3.33
Magway Region	3.80	4.21	4.56	3.33	3.29	3.40
Mandalay Region	4.68	5.14	5.58	4.02	4.19	4.20
Mon State	1.68	1.87	2.04	1.48	1.36	1.19
Rakhine State	2.28	2.47	2.65	1.75	2.95	2.06

Yangon Region	1.43	1.49	1.58	2.20	2.74	2.36
Shan State	3.77	3.93	4.16	4.43	4.90	4.79
Ayeyawady Region	5.65	6.15	6.52	5.31	5.32	4.66

Table S23. Grain consumption and production by States and Regions of Myanmar (million kg)

	Consumption				Production			
**2000**	**Rice**	**Maize**	**Wheat**	**Total**	**Rice**	**Maize**	**Wheat**	**Total**
Union	7803.11	411.03	3033.30	11247.43	21644.59	375.22	90.56	22110.36
Kachin State	205.45	10.73	78.58	294.76	565.92	10.25	0.04	576.20
Kayah State	42.92	2.27	16.83	62.02	108.17	10.94	0.08	119.19
Kayin State	243.43	12.40	88.98	344.81	661.48	1.84	0.00	663.31
Chin State	76.22	3.94	28.58	108.74	150.52	58.82	0.09	209.43
Sagaing Region	863.06	44.43	321.74	1229.22	2360.17	87.87	77.68	2525.72
Tanintharyi Region	216.25	11.38	83.94	311.57	419.07	0.10	0.00	419.17
Bago Region	808.96	42.20	308.95	1160.11	3700.31	3.62	0.00	3703.93
Magway Region	743.47	38.34	278.00	1059.81	833.02	58.59	0.65	892.26
Mandalay Region	1072.24	56.87	421.97	1551.08	1102.61	32.98	12.71	1148.30
Mon State	392.61	20.89	155.45	568.96	1067.11	0.04	0.00	1067.14
Rakhine State	447.02	23.06	167.27	637.35	1341.32	0.00	0.00	1341.32
Yangon Region	817.33	47.57	377.84	1242.73	1927.30	0.05	0.00	1927.35
Shan State	772.78	40.21	293.84	1106.83	1526.37	192.71	15.24	1734.32
Ayeyawady Region	1101.36	56.75	411.34	1569.45	6507.43	33.36	1.04	6541.83
**2005**	**Rice**	**Maize**	**Wheat**	**Total**	**Rice**	**Maize**	**Wheat**	**Total**
Union	8601.84	454.25	3359.09	12415.18	27057.12	930.08	153.83	28141.04
Kachin State	226.79	11.91	87.74	326.44	639.49	24.30	0.09	663.89
Kayah State	49.28	2.62	19.42	71.32	159.56	19.79	0.00	179.36
Kayin State	268.56	13.73	98.81	381.10	895.91	26.44	0.00	922.35
Chin State	81.95	4.25	30.94	117.13	178.21	105.60	0.12	283.93
Sagaing Region	958.59	49.43	358.47	1366.49	2989.79	211.17	113.58	3314.54
Tanintharyi Region	242.82	12.81	94.63	350.26	607.77	0.06	0.00	607.83
Bago Region	881.10	45.99	336.95	1264.04	4567.14	6.14	0.00	4573.29
Magway Region	824.53	42.53	308.56	1175.62	1209.66	100.71	0.89	1311.27
Mandalay Region	1193.61	63.46	471.81	1728.87	1575.41	49.39	17.72	1642.53
Mon State	441.19	23.52	175.21	639.91	1381.84	0.00	0.00	1381.84
Rakhine State	488.48	25.24	183.35	697.07	1657.01	0.02	0.00	1657.03
Yangon Region	906.76	52.97	421.83	1381.56	2025.79	0.02	0.00	2025.81
Shan State	831.33	43.51	319.43	1194.26	2056.21	350.25	19.98	2426.44
Ayeyawady Region	1206.87	62.28	451.94	1721.09	7113.87	35.90	1.29	7151.07
**2010**	**Rice**	**Maize**	**Wheat**	**Total**	**Rice**	**Maize**	**Wheat**	**Total**
Union	9276.72	490.20	3626.76	13393.68	32786.79	1368.36	180.39	34335.55
Kachin State	246.10	12.95	95.46	354.51	1088.37	47.37	0.17	1135.91
Kayah State	54.96	2.92	21.69	79.57	185.22	29.51	0.01	214.74
Kayin State	290.65	14.89	107.41	412.95	1119.83	55.32	0.00	1175.15
Chin State	87.57	4.54	33.11	125.22	229.09	130.46	0.15	359.70

Sagaing Region	1039.75	53.64	389.11	1482.49	3779.85	279.75	128.37	4187.98
Tanintharyi Region	265.91	14.05	104.01	383.98	612.95	0.30	0.00	613.25
Bago Region	943.58	49.27	360.98	1353.82	5662.70	2.71	0.00	5665.41
Magway Region	893.78	46.11	334.51	1274.40	1737.05	129.98	1.09	1868.11
Mandalay Region	1298.53	69.06	513.58	1881.16	1590.42	67.79	20.23	1678.44
Mon State	482.63	25.72	191.62	699.97	1660.63	0.00	0.00	1660.63
Rakhine State	524.50	27.11	196.99	748.59	2040.41	0.23	0.00	2040.64
Yangon Region	984.72	57.59	458.92	1501.23	2297.24	0.14	0.00	2297.38
Shan State	885.84	46.41	341.04	1273.28	2542.16	604.71	28.71	3175.58
Ayeyawady Region	1278.22	65.94	478.33	1822.49	8240.61	20.49	1.49	8262.60
**2015**	**Rice**	**Maize**	**Wheat**	**Total**	**Rice**	**Maize**	**Wheat**	**Total**
Union	7820.10	412.30	3044.91	11277.31	26210.32	1748.55	179.31	28138.17
Kachin State	251.99	13.46	100.50	365.95	613.59	75.79	0.04	689.42
Kayah State	44.89	2.35	17.19	64.42	121.78	58.09	0.00	179.87
Kayin State	237.63	12.33	89.92	339.89	944.96	111.05	0.00	1056.01
Chin State	75.84	3.93	28.59	108.35	79.28	38.01	0.17	117.45
Sagaing Region	849.47	43.67	315.83	1208.97	3040.04	265.14	122.02	3427.20
Tanintharyi Region	221.50	11.55	84.48	317.53	377.29	0.14	0.00	377.43
Bago Region	768.75	39.91	291.06	1099.72	4507.48	14.30	0.00	4521.78
Magway Region	627.49	32.12	231.50	891.11	1380.64	129.58	1.12	1511.35
Mandalay Region	948.65	50.55	376.53	1375.74	768.58	47.23	16.87	832.68
Mon State	320.61	16.84	124.03	461.48	992.65	0.00	0.00	992.65
Rakhine State	335.01	17.21	124.44	476.66	1516.44	1.05	0.00	1517.48
Yangon Region	1049.41	60.36	475.60	1585.38	1869.62	0.17	0.00	1869.79
Shan State	916.45	47.77	349.48	1313.70	2049.46	929.56	39.10	3018.12
Ayeyawady Region	992.59	50.72	364.99	1408.29	7647.61	49.65	0.00	7697.26
**2020**	**Rice**	**Maize**	**Wheat**	**Total**	**Rice**	**Maize**	**Wheat**	**Total**
Union	8484.19	447.55	3306.67	12238.41	25982.70	2072.48	105.46	28160.63
Kachin State	295.37	15.71	116.85	427.93	701.95	92.89	0.04	794.88
Kayah State	51.43	2.68	19.63	73.73	117.29	95.51	0.00	212.79
Kayin State	254.79	13.23	96.57	364.59	784.50	115.56	0.00	900.06
Chin State	82.64	4.28	31.16	118.08	66.30	24.54	0.02	90.86
Sagaing Region	890.25	45.83	331.91	1267.99	2697.06	242.68	68.75	3008.49
Tanintharyi Region	234.31	12.23	89.65	336.19	330.82	0.00	0.00	330.82
Bago Region	778.19	40.54	296.51	1115.25	4732.55	19.65	0.00	4752.19
Magway Region	628.50	32.26	233.04	893.80	885.93	37.23	0.17	923.34
Mandalay Region	1003.50	53.60	400.00	1457.11	970.80	53.04	4.40	1028.24
Mon State	307.46	16.28	120.61	444.34	949.06	0.01	0.00	949.07
Rakhine State	539.01	27.40	196.38	762.80	1249.95	0.02	0.00	1249.97
Yangon Region	1202.86	68.77	539.54	1811.17	1948.43	1.89	0.00	1950.32
Shan State	1011.89	52.72	385.59	1450.20	2028.77	1246.48	32.07	3307.32
Ayeyawady Region	1004.29	51.43	370.83	1426.55	8202.06	118.33	0.00	8320.39
**2025**	**Rice**	**Maize**	**Wheat**	**Total**	**Rice**	**Maize**	**Wheat**	**Total**
Union	7975.05	421.28	3116.02	11512.35	29817.28	2425.80	96.29	32339.37
Kachin State	330.67	17.65	131.69	480.02	696.23	80.71	0.02	776.96
Kayah State	46.25	2.44	17.97	66.66	105.57	87.72	0.00	193.29
Kayin State	237.05	12.32	89.99	339.37	782.32	113.62	0.00	895.94
Chin State	58.89	3.06	22.31	84.26	57.37	19.79	0.85	78.01

Sagaing Region	951.49	49.01	355.13	1355.62	3343.69	262.28	65.35	3671.32
Tanintharyi Region	219.10	11.48	84.36	314.94	346.33	0.00	0.00	346.34
Bago Region	698.78	36.52	267.84	1003.15	6015.98	49.04	0.00	6065.03
Magway Region	651.24	33.44	241.65	926.33	1079.85	43.35	0.23	1123.42
Mandalay Region	975.46	51.85	385.40	1412.71	880.08	58.31	4.37	942.76
Mon State	281.83	15.02	111.91	408.77	1028.07	0.03	0.00	1028.09
Rakhine State	401.98	20.72	150.24	572.94	1117.76	0.04	0.00	1117.79
Yangon Region	1055.79	60.43	474.59	1590.82	2156.31	2.08	0.00	2158.39
Shan State	1006.17	52.58	385.52	1444.27	1947.13	1577.85	25.48	3550.45
Ayeyawady Region	886.71	45.48	328.32	1260.50	9953.73	112.96	0.00	10066.69

Table S24. Staple food self-sufficiency at the national level in Myanmar, 2000-2025

Years	Rice	Maize	Wheat	Total grain
2000	2.77	0.91	0.03	1.97
2005	3.15	2.05	0.05	2.27
2010	3.53	2.79	0.05	2.56
2015	3.35	4.24	0.06	2.50
2020	3.06	4.63	0.03	2.30
2025	3.74	5.76	0.03	2.81

Table S25. Staple food self-sufficiency at the regional level in Myanmar, 2000-2025

Self-sufficiency ratio	2000	2005	2010	2015	2020	2025
Kachin State	1.95	2.03	3.20	1.88	1.86	1.62
Kayah State	1.92	2.51	2.70	2.79	2.89	2.90
Kayin State	1.92	2.42	2.85	3.11	2.47	2.64
Chin State	1.93	2.42	2.87	1.08	0.77	0.93
Sagaing Region	2.05	2.43	2.82	2.83	2.37	2.71
Tanintharyi Region	1.35	1.74	1.60	1.19	0.98	1.10
Bago Region	3.19	3.62	4.18	4.11	4.26	6.05
Magway Region	0.84	1.12	1.47	1.70	1.03	1.21
Mandalay Region	0.74	0.95	0.89	0.61	0.71	0.67
Mon State	1.88	2.16	2.37	2.15	2.14	2.52
Rakhine State	2.10	2.38	2.73	3.18	1.64	1.95
Yangon Region	1.55	1.47	1.53	1.18	1.08	1.36
Shan State	1.57	2.03	2.49	2.30	2.28	2.46
Ayeyawady Region	4.17	4.15	4.53	5.47	5.83	7.99
